# Regulatory network and interplay of hepatokines, stellakines, myokines and adipokines in nonalcoholic fatty liver diseases and nonalcoholic steatohepatitis

**DOI:** 10.3389/fendo.2022.1007944

**Published:** 2022-09-30

**Authors:** Bing Yang, Liqing Lu, Dongmei Zhou, Wei Fan, Lucía Barbier-Torres, Justin Steggerda, Heping Yang, Xi Yang

**Affiliations:** ^1^ Department of Geriatric Endocrinology and Metabolism, Guangxi Key Laboratory of Precision Medicine in Cardio-cerebrovascular Diseases Control and Prevention, Guangxi Clinical Research Center for Cardio-cerebrovascular Diseases, The First Affiliated Hospital of Guangxi Medical University, Nanning, China; ^2^ Department of Thoracic Surgery, Xiangya Hospital, Central South University, Changsha, China; ^3^ Division of Digestive and Liver Diseases, Cedars-Sinai Medical Center, Los Angeles, CA, United States; ^4^ Department of Surgery, Cedars-Sinai Medical Center, Los Angeles, CA, United States

**Keywords:** hepatokines, stellakines, myokines, adipokines, nonalcoholic fatty liver disease, nonalcoholic steatohepatitis

## Abstract

Fatty liver disease is a spectrum of liver pathologies ranging from simple hepatic steatosis to non-alcoholic fatty liver disease (NAFLD), non-alcoholic steatohepatitis (NASH), and culminating with the development of cirrhosis or hepatocellular carcinoma (HCC). The pathogenesis of NAFLD is complex and diverse, and there is a lack of effective treatment measures. In this review, we address hepatokines identified in the pathogenesis of NAFLD and NASH, including the signaling of FXR/RXR, PPARα/RXRα, adipogenesis, hepatic stellate cell activation/liver fibrosis, AMPK/NF-κB, and type 2 diabetes. We also highlight the interaction between hepatokines, and cytokines or peptides secreted from muscle (myokines), adipose tissue (adipokines), and hepatic stellate cells (stellakines) in response to certain nutritional and physical activity. Cytokines exert autocrine, paracrine, or endocrine effects on the pathogenesis of NAFLD and NASH. Characterizing signaling pathways and crosstalk amongst muscle, adipose tissue, hepatic stellate cells and other liver cells will enhance our understanding of interorgan communication and potentially serve to accelerate the development of treatments for NAFLD and NASH.

## Introduction

Nonalcoholic fatty liver disease (NAFLD) refers to hepatic steatosis >5% when not caused by excessive drinking, hepatitis, liver disease, drug use, etc. (1). NAFLD is a multisystem disease, covering a spectrum of histologies from simple steatosis, hepatic fibrosis to nonalcoholic steatohepatitis (NASH), liver cirrhosis and hepatocellular carcinoma (HCC) (2). The global prevalence of NAFLD is gradually increasing, posing a serious threat to human life and health, and there is an urgent need for new methods to both prevent and manage the disease. Current studies have found that obesity, hyperglycemia, dyslipidemia, hypertension, type 2 diabetes mellitus (T2DM), and genetic factors play pathogenic roles in the development of NAFLD. Furthermore, inflammation and insulin resistance (IR) are considered risk factors for the development of NAFLD (3).

**Figure 1:**
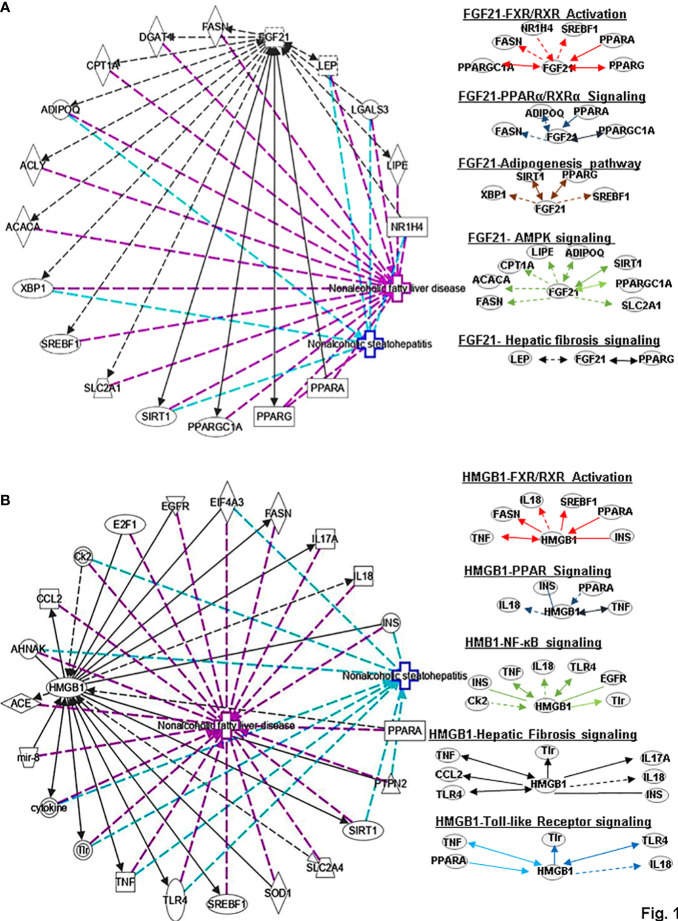
Ingenuity pathway analysis (IPA) of FGF21 and HMGB1. **(A)** IPA investigated the molecular pathways of FGF21 involved in NAFLD and NASH using the website: www.ingenuity.com. Purple lines show these genes and molecules are related to NAFLD. Green lines show these genes and molecules are associated with NASH (left). All identified pathways of FGF21 were screened individually. Representative canonical pathways of FGF21 are associated with other related genes (including the transcription regulator and enzymes, et al) and molecules of NAFLD and NASH. Functional relationships of these genes, molecules and FGF21 are depicted using straight lines with arrows. FGF21 is functionally related to 1. FXR/RXR signaling; 2. PPARα/RXRα signaling; 3. Adipogenesis signaling; 4. AMPK signaling and 5. Hepatic fibrosis signaling (Right). **(B)** IPA investigated the molecular pathways of HMGB1 involved in NAFLD and NASH. All identified pathways of HMGB1 were screened individually (left). Representative canonical pathways of HMGB1 are associated with other related genes and molecules of NAFLD and NASH. Functional relationships of these genes and HMGB1 are depicted using straight lines with arrows. HMGB1 is functionally related to: 1. FXR/RXR signaling; 2. PPAR signaling; 3. NF-κB signaling; 4. Hepatic fibrosis signaling and 5. Toll-like Receptor signaling (Right). Solid lines show direct regulation while dotted lines depict indirect interactions. PPARGC1A, PPARG Coactivator 1 Alpha; FASN, Fatty Acid Synthase; NR1H4, Nuclear Receptor Subfamily 1 Group H Member 4; SREBF1, Sterol regulatory element-binding transcription factor 1; PPARA, Peroxisome Proliferator Activated Receptor Alpha; PPARG, Peroxisome proliferator- activated receptor gamma; ADIPOQ, Adiponectin; XBP1, X-Box Binding Protein 1; SIRT1, Sirtuin 1;ACACA, Acetyl-CoA Carboxylase Alpha; CPT1A, Carnitine Palmitoyl transferase 1A; LIPE, Lipase E; SLC2A1, Solute Carrier Family 2 Member 1, LEP, Leptin; IL-18, Interleukin 18; INS, Insulin; TNF, Tumor necrosis factor; Ck2, Casein kinase II; TLR4, Toll like receptor 4; EGFR, Epidermal growth factor receptor; TIr, Toll/interleukin-1 receptor-like protein; CCL2, C-C Motif chemokine ligand 2; IL-17A, Interleukin 17A.

**Figure 2:**
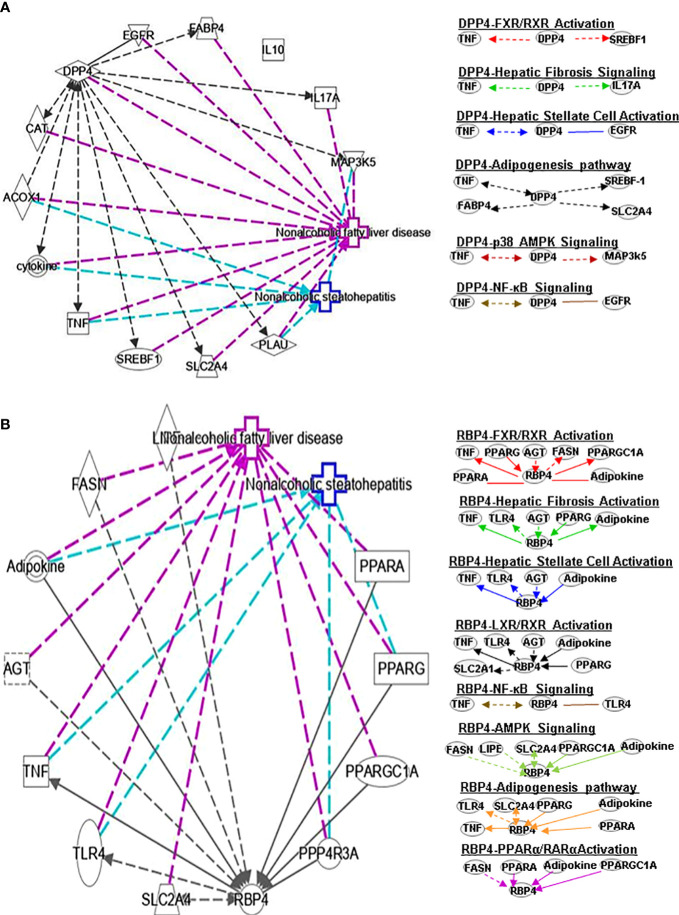
Ingenuity pathway analysis (IPA) of DPP4 and RBP4. **(A)** IPA investigated the molecular pathways of DPP4 involved in NAFLD and NASH. All identified pathways of RBP4 were screened individually. Representative canonical pathways of RBP4 are associated with other related genes and molecules of NAFLD and NASH. Functional relationships of these genes, molecules and DPP4 are depicted using straight lines with arrows. DPP4 is functionally related to 1. FXR/RXR signaling; 2. Hepatic fibrosis signaling; 3. Hepatic stellate cell activation; 4. Adipogenesis pathway; 5. p38-AMPK signaling and 6. NF-κB signaling (Right). **(B)** IPA investigated the molecular pathways of RBP4 involved in NAFLD and NASH. All identified pathways of RBP4 were screened individually (left). Representative canonical pathways of RBP4 are associated with other related genes and molecules of NAFLD and NASH. Functional relationships of these genes and RBP4 are depicted using straight lines with arrows. RBP4 is functionally related to 1. FXR/RXR signaling; 2. Hepatic fibrosis signaling; 3. Hepatic stellate cell activation; 4. LXR/RXR pathway and 5. NF-κB signaling (Right). Solid lines show direct regulation while dotted lines depict indirect interactions. SLC2A4, Solute carrier family 2 member 4; MAP3k5, Mitogen-activated protein kinase kinase kinase 5; AGT, Angiotensinogen.

**Figure 3:**
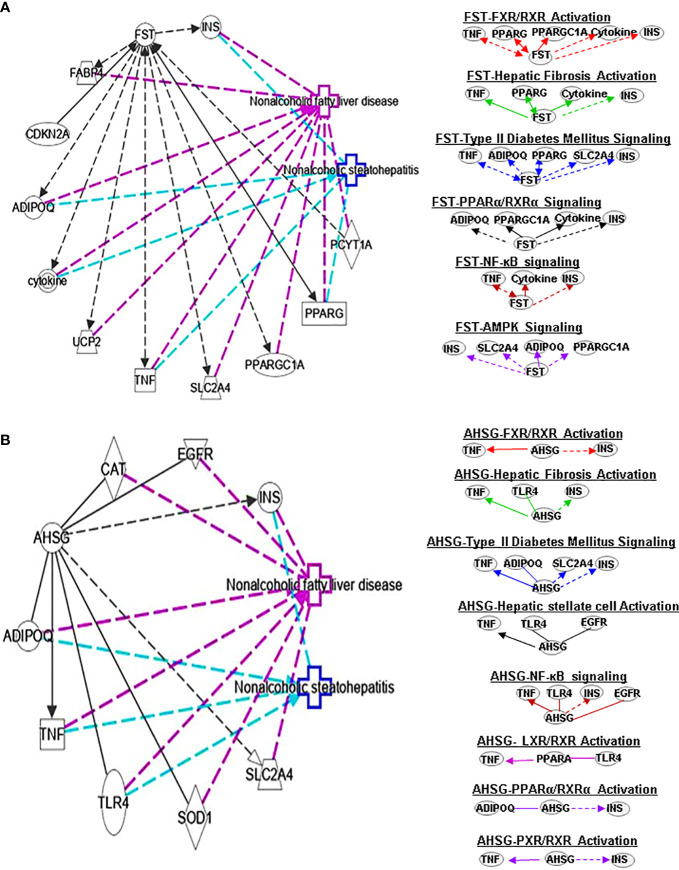
Ingenuity pathway analysis (IPA) of FST and AHSG. **(A)** IPA investigated the molecular pathways of FST involved in NAFLD and NASH. All identified pathways of FST were screened individually. Representative canonical pathways of FST are associated with other related genes and molecules of NAFLD and NASH. Functional relationships of these genes, molecules and FST are depicted using straight lines with arrows. FST is functionally related to 1. FXR/RXR signaling; 2. Hepatic fibrosis signaling; 3. Type 2 Diabetes Mellitus signaling; 4. PPARα/RXRα signaling; 5. AMPK signaling and 6. NFκB signaling (Right). **(B)** IPA investigated the molecular pathways of AHSG involved in NAFLD and NASH. All identified pathways of AHSG were screened individually (left). Representative canonical pathways of AHSG are associated with other related genes and molecules of NAFLD and NASH. Functional relationships of these genes and AHSG are depicted using straight lines with arrows. AHSG is functionally related to 1. FXR/RXR signaling; 2. Type 2 Diabetes Mellitus signaling; 3. Hepatic stellate cell activation signaling; 4. NF-κB signaling; 5. LXR/RXR signaling; 6. PPARα/RXRα signaling and 7. PXR/RXR signaling (Right). Solid lines show direct regulation while dotted lines depict indirect interactions. PPARGC1A, Peroxisome proliferator-activated receptor gamma coactivator 1-alpha.

**Figure 4:**
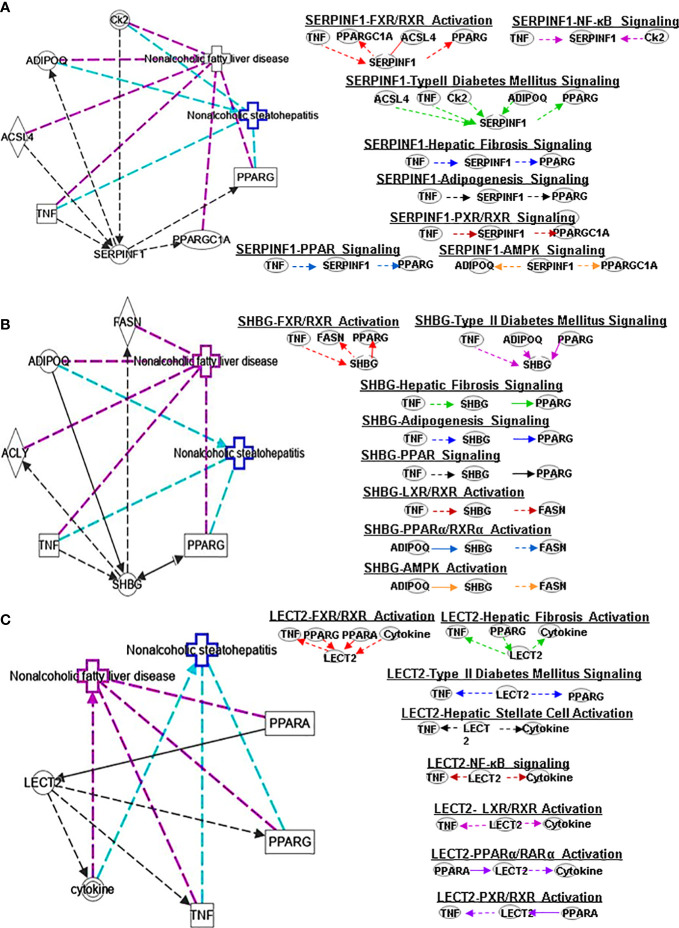
Ingenuity pathway analysis (IPA) of SERPINF1, SHBG and LECT2. **(A)** IPA investigated the molecular pathways of SERPINF1 involved in NAFLD and NASH. All identified pathways of SERPINF1 were screened individually. Representative canonical pathways of SERPINF1 are associated with other related genes and molecules of NAFLD and NASH. Functional relationships of these genes, molecules and SERPINF1 are depicted using straight lines with arrows. SERPINF1 is functionally related to 1. FXR/RXR signaling; 2. NF-κB signaling; 3. Type 2 Diabetes Mellitus signaling; 4. Hepatic fibrosis signaling; 5. Adipogenesis signaling; 6. LXR/RXR signaling; 7. PPARα/RXRα signaling and 8. AMPK signaling (Right). **(B)** IPA investigated the molecular pathways of SHBG involved in NAFLD and NASH. All identified pathways of SHBG were screened individually (left). Representative canonical pathways of SHBG are associated with other related genes and molecules of NAFLD and NASH. Functional relationships of these genes and SHBG are depicted using straight lines with arrows. SHBG is functionally related to 1. FXR/RXR signaling; 2. Type 2 Diabetes Mellitus signaling; 3. Hepatic fibrosis signaling; 4. Adipogenesis signaling; 5. LXR/RXR signaling; 6. PPARα/RXRα signaling and 7. AMPK signaling (Right). **(C)** IPA investigated the molecular pathways of LECT involved in NAFLD and NASH. All identified pathways of LECT were screened individually (left). Representative canonical pathways of LECT are associated with other related genes and molecules of NAFLD and NASH. Functional relationships of these genes and LECT are depicted using straight lines with arrows. LECT is functionally related to 1. FXR/RXR signaling; 2. Hepatic fibrosis signaling; 3. Type 2 Diabetes Mellitus signaling; 4. Hepatic stellate cell signaling; 5. NF-κB signaling; 6. LXR/RXR signaling; 6. PPARα/RXRα signaling and 7. PXR/RXR signaling (Right). Solid lines show direct regulation while dotted lines depict indirect interactions.

**Figure 5:**
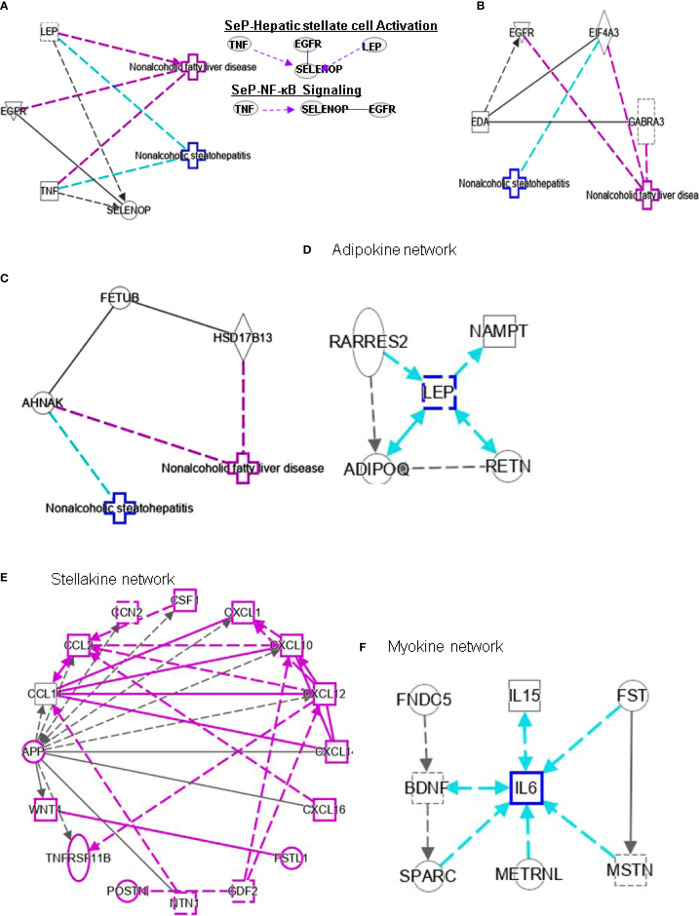
Ingenuity pathway analysis (IPA) of SELENOP, EDA and FETUB. **(A)** IPA investigated the molecular pathways of SELENOP involved in NAFLD and NASH. All identified pathways of SELENOP were screened individually. Representative canonical pathways of SELENOP are associated with other related genes of NAFLD and NASH. Functional relationships of these genes and SELENOP are depicted using straight lines with arrows. SELENOP is functionally related to 1. Hepatic stellate cell signaling and 2. NF-κB signaling (Right). **(B, C)** IPA investigated the molecular pathways of EDA and FETUB involved in NAFLD and NASH. All identified pathways of EDA and FETUB were screened individually. **(D–F)** IPA investigated the molecular pathways of Adipokine, Stellakine and Myokine network.

NAFLD can develop into NASH, which increases the risk for the development of liver fibrosis, cirrhosis, and HCC. Hepatokines (proteins secreted by hepatocytes) can affect the pathogenesis of IR and NAFLD through autocrine, paracrine, and endocrine signal transduction (4). Such proteins include fibroblast growth factor 21 (FGF21), high-mobility group box 1 (HMGB1), dipeptidyl peptidase 4 (DPP4),retinol-binding protein 4 (RBP4), follistatin (FST), alpha 2-HS glycoprotein (ASHG or FETUA), serpin Family F Member 1 (SERPINF1), sex hormone binding globulin (SHBG), leukocyte cell derived chemotaxin 2 (LECT2), tsukushi, (small leucine rich proteoglycan, TSK), angiopoietin like 4 (ANGPTL4), selenoprotein P (SeP or SELENOP), ectodysplasin A (EDA) and fetuin B (FETUB). Ingenuity pathway analysis (IPA) was used to analyze the canonical pathways by which hepatokines participate in NAFLD and NASH pathogenesis. We found that these hepatokines are involved in NAFLD and NASH progression through the following signaling pathways: farnesoid x receptor (FXR)/retinoic X receptor (RXR); pregnane X receptor (PXR)/RXR; liver X receptor (LXR)/RXR; hepatic stellate cell (HSC) activation; fibrogenesis; adipogenesis; nuclear factor - kappa B (NF-κB); peroxisome proliferator-activated receptors (PPAR); PPARα/retinoic acid receptor alpha (RARα); AMP-activated protein kinase (AMPK) and T2DM (**Figures 1**–**5**).

To classify the interplay between hepatokines, myokines, adipokines, and stellakines in NAFLD and NASH, we analyzed cell-cell signaling and interactions using IPA (**Tables 1**–**3**). Although this analysis describes the crosstalk between hepatokines, myokines, adipokines and stellakines from hepatocytes, skeletal muscle, adipocytes, and HSC, respectively in a limited manner, it will provide a better understanding of the molecular mechanisms involved in the progression of these diseases.

**Table 1:** IPA analysis of hepatokines related to NAFLD and NASH.

Hepatokine/Target	Cell-cell signaling and interaction	Metabolic action	Contribution to NAFLD and NASH
**FGF21** /Adipose tissue, brain.	Stimulation of Kupffer cells;	Hepatic steatosis; Insulin resistance; Impaired glucose tolerance; Hypertriglyceridemia.	Improve of insulin sensitivity; Prevention of HSC and liver fibrosis; Reduction of inflammation and lipid accumulation.
**HMGB1** /Liver, lung.	Recruitment, activation, adhesion of macrophages and monocytes;	Insulin resistance; Non-insulin-dependent diabetes mellitus.	Prevention of lipid metabolism disorders by maintaining β-oxidation and preventing ER stress; Inhibition of HNF1A to regulate hepatic fibrosis.
**DPP4** /Liver, adipocytes, HSC.	Binding of extracellular and activation of monocytes	Nonalcoholic fatty liver disease; Insulin-dependent diabetes mellitus; Post transplant diabetes mellitus.	Promotion of inflammation, IR, and fibrosis.
**RBP4** /Live, adipocytes.	Activation of antigen presenting cells	Non-insulin-dependent diabetes mellitus.	Diagnosis of advance fibrosis
**FST**/Peripheral tissues.	Unknown	Hyperinsulinemia Impaired glucose tolerance.	Progression from simple steatosis to NASH; Inhibition of HSC proliferation and fibrosis.
**ASHG** /Liver, skeletal Muscle.	Adhesion of tumor cells	Insulin sensitivity. Glucose tolerance.	Inhibition of HSC proliferation, and differentiation into myofibroblaster.
**SERPINF1** /Liver, adipose Tissue.	Activation of pancreatic stellate cells, Activation of macrophages	Non proliferative diabetic retinopathy with macular edema.	Prevention of liver fibrosis and HSC activation
**SHBG** /Androgens, estrogens.	Unknown	Hyperinsulinism; Accumulation of triacylglycerol.	Prevention of liver fibrosis and HSC activation
**LECT2** /Skeletal muscle, liver, adipose tissue.	Activation of macrophages	Liver fibrosis and cirrhosis	Involvement of liver steatosis, and inflammation; Progression of the liver from simple steatosis to NASH.
**SELENOP** /peripheral Tissues.	Unknown	lower SeP levels in patients with NASH.	Association between high SeP level and a lower risk of HCC
**EDA** /Live, hair and teeth.	Unknown	systemic insulin sensitivity, and steatosis	Unknown
**FETUB** /Live and ovaries.	Unknown	Induction of IR; Aggravation of liver steatosis	Unknown

Ingenuity pathway analysis (IPA) analysis of hepatokines related to NAFLD and NASH. Screen all identified hepatokines individually. Representative cell–cell signaling and interaction of hepatokines are associated with metabolic liver damage and expression levels in NAFLD and NASH.

**Table 2:** IPA analysis of Cell-cell signaling/interaction of adipokine and interplay with hepatokines.

Adipokine	Cell-cell signaling and interaction	Interaction between adipokine and hepatokines
**ADIPOQ**	Activation of hepatic stellate cells; Recruitment, attachment, and binding macrophages; Activation of monocytes.	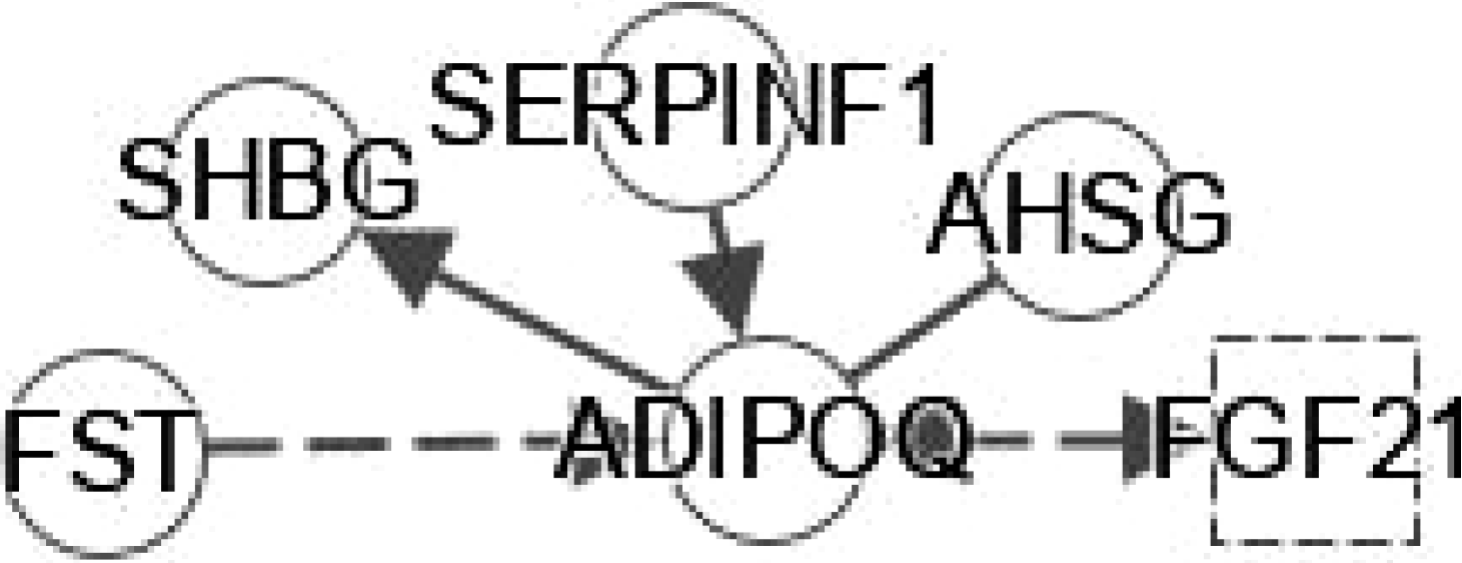
**LEP**	Activation of hepatic stellate cells; Binding of hepatocytes; Oxidative stress response of endothelial cells.	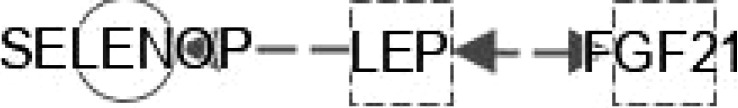
**NAMPT**	Autophagy of cytoplasm; Polarization of M2 macrophages	Undetected
**RETN**	Migration and proliferation of hepatic stellate cell; Insulin sensitivity of liver.	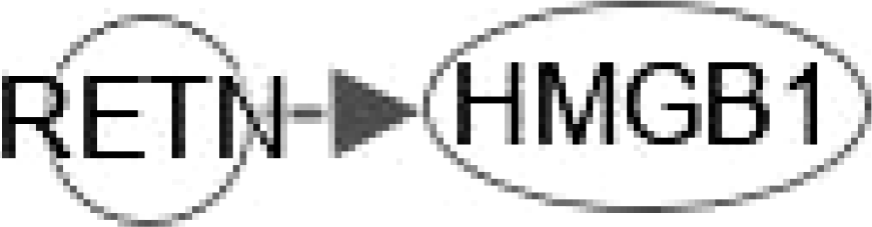
**RARRES2**	Attachment of vascular endothelial cells; Recruitment of monocytes and neutrophils; Uptake of fatty acid.	Undetected
**RBP4**	Insulin tolerance; Non-insulin-dependent diabetes mellitus	Undetected
**FNDC5**	Alzheimer disease	Undetected

Ingenuity pathway analysis (IPA) of adipokines. All identified disease and function of adipokines were screened individually. Representative cell-cell signaling and interaction of stellakines are associated with disease andfunction. ADIPOQ, Adiponectin; Lep, Leptin; NAMPT, nicotinamide phosphoribosyltransferase; RETN, Resistin; RARRES2, retinoic acid receptor responder 2; RBP4, Retinol-binding protein 4 and FNDC5, fibronectin type III domain containing 5.

**Table 3:** IPA analysis of Cell-cell signaling and interaction of HSC-derived stellakines.

Stellakine	Cell-cell signaling and interaction	Interaction between adipokine and hepaokines
**APP**	Oxidative stress response of endothelial cells; Targeting of macrophages Targeting of T lymphocytes	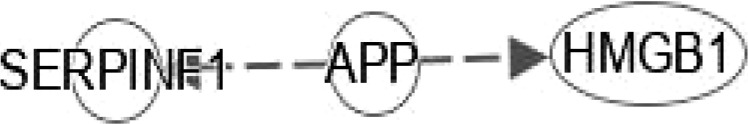
**CCL2 **	Recruitment of Kupffer cell, inflammatory cell; Cytokine and chemokine mediated signaling pathway	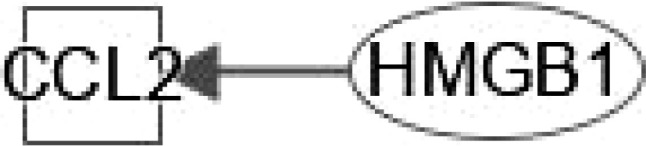
**CCL11**	Activation of macrophages and monocytes; Cytokine and chemokine mediated signaling pathway Aggregation and adhesion of T lymphocytes	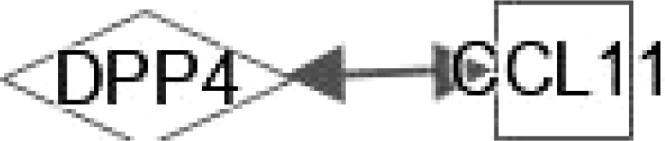
**CSF1**	Activation of Kupffer cells Induction of M2 macrophages Recruitment of monocytes	Undetected
**CTGF**	Adhesion of hepatic stellate cells Attachment of epithelial cell lines Adhesion of cell-associated matrix Induction of extracellular matrix	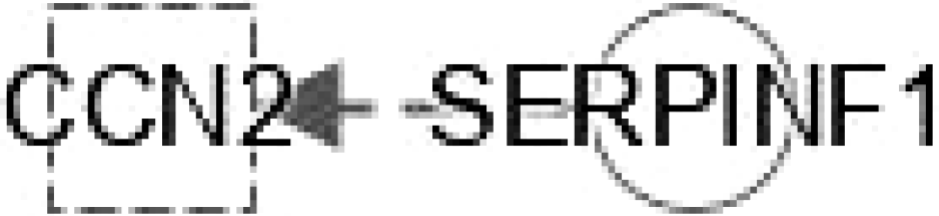
**CXCL1**	Adhesion of endothelial cells Recruitment, activation and chemoattraction of neutrophils Cytokine and chemokine mediated signaling pathway	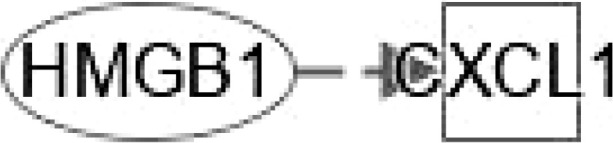
**CXCL10** ** **	Recruitment of inflammatory leukocytes Chemoattraction of monocytes Cytokine and chemokine mediated signaling pathway; Recruitment of monocyte-derived macrophages Activation of endothelial cells	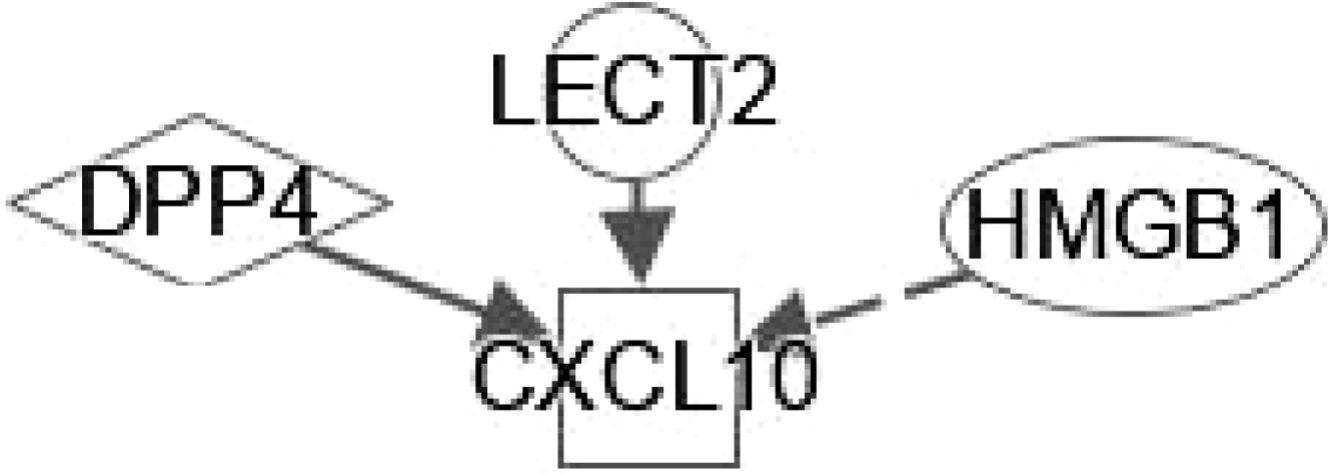
**CXCL12**	Activation of hepatic stellate cells Recruitment of fibrocytes Initiation of chemoattraction of cells	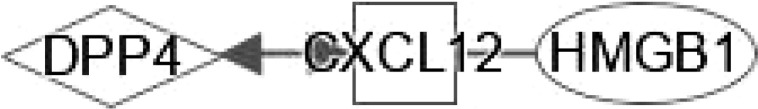
**CXCL14**	Chemoattraction of neutrophils	Undetected
**CXCL16**	Attraction of cells; Cell-cell adhesion	Undetected
**GAS6**	Adhesion of endothelial cells Adhesion of neutrophils	
**NTN1**	Inflammatory response of macrophage Recruitment of neutrophils Cell-cell adhesion	Undetected
**POSTN**	Adhesion of cell-associated matrix	Undetected
**WNT4**	Activation of hepatic stellate cells, Cell-cell adhesion	Undetected

Ingenuity pathway analysis (IPA) of stellakines. All identified disease and function of stellakines were screened individually. Representative cell-cell signaling and interaction of stellakines are associated with disease and function. APP, Amyloid Beta Precursor Protein; CCl2, C-C motif chemokine ligand 2; CCL11, C-C motif chemokine ligand 11; CSF1, Colony stimulating factor 1; CTGF, Connective tissue growth factor; CXCL1, C-X-C motif chemokine ligand 1; CXCL10, C-X-C motif chemokine ligand 10; CXCL12, C-X-C motif chemokine ligand 12; CXCL14, C-X-C motif chemokine ligand 14; CXCL16, C-X-C motif chemokine ligand 14; CXCL16, C-X-C motif chemokine ligand 16; GAS6, Growth arrest-specific gene 6; NTN1, Netrin 1; POSTN, Periostin and WNT4, Wnt Family Member 4.

## Hepatokines and FXR/RXR, PPARα/RXRα signaling pathways

FXR forms a heterodimeric complex with RXR (FXR/RXR), which plays a significant role in glucose and lipid homeostasis. As shown in **Figures 1**–**5**, several hepatokines can affect metabolism through the FXR/RXR signaling pathway in NAFLD and NASH. Previous studies have found that PPARα/RXRα signaling can participate in human metabolic diseases by regulating oxidative stress, lipid metabolism, and inflammatory pathways, which suggests that it is possible that hepatokines influence the development of NAFLD and NASH *via* PPARα/RXRα signaling pathway.

FGF21 is a liver protein which regulates glucose homeostasis and metabolism. Moreover, the levels of FGF21 have a positive correlation with triglycerides (TGs), fasting insulin and IR (5). The canonical pathways associated with NAFLD and NASH analysis also indicated that FGF21 can regulate the related genes peroxisome proliferator-activated receptor gamma (PPARG), peroxisome proliferator-activated receptor alpha (PPARA), sterol regulatory element binding protein 1 (SREBF1), farnesoid X receptor (NRIH4), fatty acid synthase (FAS or FASN), and peroxisome proliferator activated receptor gamma coactivator 1 alpha (PPARGC1A) by affecting FXR/RXR signaling to play a role in metabolic diseases (**Figure 1A**). FGF21 can also regulate the lipolysis of white adipose tissue. Furthermore, PPARα is a key mediator of liver FGF21 expression and function, including the regulation of gluconeogenesis, ketone body production, numbness, and growth inhibition. Hepatic JNK signaling pathway deficiency promotes the repression of PPARα target gene FGF21 expression by regulating corepressor (NCoR1 and NRIP1) function. PPARγ activity can affect part of FGF21 and regulate adiponectin transcription and secretion in adipocytes. In addition, FGF21 has been found to play a potential role in treating NASH by inhibiting inflammation and fibrosis, reducing fatty liver and liver cell damage. The expression of FGF21 is controlled by RXR/FXR and can also be induced by all-trans retinoic acid (ATRA) activation of RARα and RARβ (6). FASN plays a central role in *de novo* lipogenesis. Adiponectin (ADIPOQ) is expressed exclusively in adipose tissue. PPARα seems to act as a sensor for the homeostasis of fatty-acid metabolism and modulates the responses in the liver, kidney, and heart. Peroxisome proliferator-activated receptor γ coactivator 1-α (PGC1α) is a coactivator of nuclear receptors and other transcription factors that regulate metabolic processes, including mitochondrial biosynthesis and respiration, liver gluconeogenesis and muscle fiber type switching. IPA analysis for FGF21- PPARα/RXRα signaling in NAFLD, and NASH showed that FGF21 interacts with these genes including FASN, ADIPOQ, PPARA and PPARGC1A (**Figure 1A**).

HMGB1 plays important roles in regulating the pathogenesis of NAFLD. HMGB1 promotes human embryonic lung fibroblast (HLF-1) proliferation and extracellular matrix (ECM) production by activating HLF-1-α-regulated aerobic glycolysis (7). Sirtuin 1 (SIRT1) plays an essential role in the pathophysiology of NAFLD. SIRT1 regulates the transcriptional activity of PPARRα and PGC1α (PPARα coactivator1α) in the liver (8–10) to increase the expression of genes involved in oxidation, thereby increasing FAO. Conversely, liver-specific deletion of SIRT1 results in FAO blunting by inhibiting the induction of PPAR target genes (11). Circulating levels of SIRT4 are low in obese patients with NAFLD and negatively regulates mitochondrial oxidative metabolism in primary hepatocytes and myotubes (12, 13). Nasrin et al. (12)found that SIRT4 knockdown or inhibition resulted in increased AMPK activity, while AMPK activation increased NAD levels, thereby promoting SIRT1 targets such as PGC-1α and FOXO proteins) (14, 15),and, SIRT1-mediated activation of AMPK (by deacetylating LKB1) increases ACC phosphorylation, resulting in increased FAO (12). However, we need a comparative post-assessment of SIRT4 expression or activity in hepatocyte or muscle cell cultures (13).High glucose inhibits protein kinase A (PKA)-induced insulin-like growth factor binding protein 3 (IGFBP-3) and SIRT1 and increases cytoplasmic HMGB1 in retinal endothelial cells (REC) (16). Some findings indicated that HMGB1 in hepatocytes prevents lipid metabolism disorders by maintaining β-oxidation and preventing endoplasmic reticulum (ER) stress, which suggests that stabilizing HMGB1 in liver cells may become an effective way to prevent and treat NAFLD. Tumor necrosis factor (TNF) is a core pathogenic factor in NAFLD. Interleukin-18 (IL-18) is a proinflammatory cytokine associated with metabolic syndrome (MS), of which is often considered as a feature of NAFLD. Sterol regulatory element–binding protein 1 (SREBP1) has been reported to extensively regulate the key enzymes involved in synthesizing fatty acids (FA), such as FASN, acetyl-coenzyme A carboxylase (ACC) and stearoyl-CoA desaturase (SCD1). The signaling pathway IPA shows that HMGB1 can interact with genes such as TNF, FASN, IL-18, SREBF1, PPARA, and INS (insulin gene) in FXR/RXR signaling (**Figure 1B**). In addition, HGBM1 interacts with IL18, INS, PPARA and TNF in PPARα/RXRα signaling (**Figure 1B**). Altogether these results indicate that HMGB1 is playing a prominent role in the pathogenesis of NAFLD and NASH.

DPP4 is a 110 kDa serine protease expressed on the surface of a variety of cells. DPP4 plays an important role in regulating human glucose metabolism. In tissues, it mainly acts by rapidly inactivating circulating glucagon-like peptide (GLP)-1 and gastric inhibitory peptide (GIP), which are mainly secreted in the liver (17). The main function of GLP-1 and GIP is to promote the release of insulin and regulate tissue glucose metabolism of tissues in the human body. Furthermore, it has been shown that DPP4 promotes inflammation and IR in obesity. DPP4 interacts with TNF and SREBF1 in FXR/RXR signaling (**Figure 2A**) and may play a key role in bile acid regulation and glucose and lipid homeostasis.

RBP4 is a serum transporter of vitamin A. Liver cells and adipose tissue are the main RBP4 secretion sites in the human body. The level of RBP4 is downregulated in advanced NAFLD compared to mild disease. Activation of c-Jun N-terminal kinase (JNK) and Toll-like receptor 4 (TLR4) signaling can cause overexpression of RBP4, leading to inflammation and IR in mouse adipose tissue (18). In contract, RBP4 gene deletion can enhance insulin sensitivity (19) and a negative correlation between the expression level of serum RBP4 and the severity of liver dysfunction has been observed. A longitudinal study suggested that RBP4 levels are associated with the development and regression of NAFLD and that they are also an independent predictor of NAFLD progression (20). Some studies found that PPARα can regulate RBP4 gene expression. Rosell et al. concluded that PPARγ and PPARα mediate RBP4 gene expression and release in brown adipose tissue, and that thermogenesis activation induces RBP4 gene expression in brown fat through a mechanism involving PGC1α signaling (21). IL-6 regulates RBP4 transcription through PPARα and CCAAT/enhancer binding protein (22). RBP4 can indirectly regulate RAR or RXR by controlling the transport of vitamin A (23). RBP4 also interacts with PPARA, TNF, PPARG, angiotensinogen (AGT), FASN and PPARGC1A genes in the FXR/RXR signaling pathway (**Figure 2B**).

FST is a member of the TNF-β family, which exists in various tissues in the human body. It also can improve islet function and regulate glucose homeostasis. Uncoupling protein 1 (UCP1), an integral membrane protein found in the mitochondrial inner membrane of brown adipose tissue, can facilitate the process of non-shivering thermogenesis in mammals. Myogenic factor 5 (MYF5) is a sticking point protein that regulates muscle differentiation, myogenesis and skeletal muscle development. FST promotes UCP1 expression by increasing mitogen-activated protein kinases (MAPK)/extracellular-signal-regulated kinase (ERK)1/2 protein phosphorylation in white adipose tissue and MYF5 protein expression in brown adipose tissue (24). Thus, circulating FST may be used as a marker of the glucagon-to-insulin tone (25). It is also well known that FST promotes IL-1β (a proinflammatory cytokine) expression. FST has antiapoptotic and antioxidant effects and regulates glucose metabolism and lipid metabolism. This indicates a potential connection between FST and PPARα/RXRα signaling. Interestingly, forkhead box O1 -mediated transcriptional activation may lead to increased serum FST levels in NAFLD patients (26). It has also been found that FST may promote the progression from simple steatosis to NASH (27). Studies have found that PPARγ downregulates FST through SP1 to delay the progression of NASH, which may offer an opportunity for a potential treatment for NASH. FST can further interplay with TNF, PPARG, ADIPOQ, PPARGC1A, cytokines and INS in FXR/RXR signaling (**Figure 3A**).

α2-HS-glycoprotein (AHSG, also known as FETUA) is one of the most important hepatokines in the human body and regulates metabolic processes, such as IR, glucose homeostasis, lipid metabolism and NAFLD. AHSG is produced mainly in the liver. Insulin receptor substrate 1 (IRS1) can transmit signals from insulin and insulin-like growth factor 1 receptors to the intracellular PI3K/AKT and ERK MAP kinase pathways. AHSG was shown to be an inhibitor of IRS1. AHSG also participates in regulating insulin sensitivity. AHSG can inhibit insulin receptor tyrosine resulting in IR; however, it is also reduced by melatonin which in turn improves IR and liver steatosis (28). The expression of AHSG is variable in patients with NAFLD. Some studies show AHSG expression in increased with advanced disease compared to mild disease. More research into the mechanisms controlling AHSG expression is needed to understand its role in NAFLD/NASH pathogenesis. AHSG interacts with TNF, ADIPOQ and INS in FXR/RXR signals (**Figure 3B**).

PEDF is a 50-kDa protein that is secreted by human fetal pigment epithelial cells and is encoded by the SERPINF1 gene. Chung et al. (29) found that PEDF affects lipid metabolism by preventing the accumulation of intracellular TGs through adipose triglyceride lipase (ATGL). SERPINF1 levels were significantly increased in patients with NAFLD. However, SERPINF1 level decreased in patients with advanced NAFLD compared with mild disease. The variable levels of SERPINF1 expression were also observed in patients with NASH. SERPINF1 has a reciprocal relationship with TNF, PPARGC1A, ACSL4 and PPARG in FXR/RXR signaling (**Figure 4A**)

SHBG is a hepatokine primarily secreted by the liver that can regulate metabolism. Compared with healthy patients, individuals with hepatic steatosis have lower levels of SHBG in the liver (30) and serum (31). A separate study also found that the serum SHBG concentration correlated with the occurrence and regression of NAFLD (32). SHBG is a new sign of metabolism and inflammation and low SHBG could predict the development of metabolic syndrome. Decreased SHBG may lead to obesity-related endocrine mechanisms and chronic inflammation (33). The proinflammatory cytokine IL-1β (34) may reduce SHBG expression mediated *via* hepatocyte nuclear factor 4 α (HNF4α). A later study showed that SHBG is negatively correlated with NAFLD in elderly individuals in China (30). Low plasma SHBG levels are associated with obesity, IR, NAFLD and T2DM. IPA shows the interplay of SHBG and the genes ADIPOQ and FASN in PPARα/RXRα signaling (**Figure 4B**).

LECT2 was primarily identified as a hepatocyte-secreted chemokine-like factor. Recent studies found positive correlations between serum levels of LECT2 and obesity, the severity of liver steatosis and IR in both mouse models and humans (35). LECT2 levels were found significantly higher in lives of patients with NAFLD. Moreover, the expression of LECT2 decreases in tissue inflammation and fibrosis, which suggests that LECT2 plays a positive role under normal conditions. Mammalian target of rapamycin (MTOR) phosphorylation, sterol regulatory element-binding protein (SREBP-1) cleavage and lipid accumulation in hepatocytes can be induced by LECT2 through a JNK-dependent mechanism (36). These results suggest that LECT2-mediated hepatic steatosis can regulate the development of NAFLD and NASH. The expression of LECT2 decreases in tissue inflammation and fibrosis, which suggest that LECT2 plays a positive role under normal conditions. Jung et al. (37) found that inflammation and IR of adipocytes are stimulated by LECT2 through the activation of CD209/P38-dependent pathways. Furthermore, Takata et al. (38) were found that LECT2 influences the development of liver inflammation and the progression of steatosis to NASH. LECT2 could induce the occurrence and development of NAFLD through the activators of transcription-1(STAT-1) pathway (39). IPA indicates that LECT2 interacts with TNF, PPARG, PPARA and cytokines in FXR/RXR signaling (**Figure 4C**).

Selenium is mainly transported from the liver to extrahepatic tissues by selenoprotein P (SeP or SELENOP). Circulating SELENOP levels are increased in subjects with NAFLD as well as in those with visceral obesity (40). Elevated circulating SELENOP concentrations were associate with glucose metabolism dysregulation and were related to IR and inflammation. Lower SELENOP levels in patients with definite NASH may be the downregulation of SELENOP in this condition characterized by liver inflammation. Leptin (LEP) is an adipocyte hormone that maintains homeostatic control of adipose tissue mass and functions. Interplay of SELENOP, TNF, EGFR and LEP are involved in FXR/RXR signaling (**Figure 5A**).

EDA belongs to a type II transmembrane protein substance of the TNF superfamily (41). EDA is considered a type of heparin that can cause systemic insulin sensitivity by activating the skeletal muscle inflammatory system in obese patients (42). Bayliss et al. found that EDA increased in NAFLD and was not related to T2DM (43). In addition, the latest research shows that plasma EDA is increased in NAFLD and NASH, which is related to steatosis, but it is not a reliable biomarker of NASH. However, EDA aggravates steatosis by striking a balance between lipid deposition and elimination, indicating that it is a potential biomarker for NAFLD (41). Levels of fetuin B are related to IR (44) and are increased in humans with steatosis (44, 45). The study demonstrated the role of fetuin-B in liver fat accumulation in IR in humans. It was also shown that FETUB might link NAFLD to chronic kidney disease by inducing IR (46). In addition, a study showed that liver steatosis can be mediated by fetuin B through the AMPK pathway to aggravate liver X receptors (47). There are no reports for the interplay between EDA or FETUB and other genes in FXR/RXR signaling (**Figures 5B, C**).

Hepatokine involvement through FXR/RXR signaling pathway in NAFLD and NASH includes FGF21, HMBG1, DPP4, RBP4, FST, AHSG, SERPINF, SHBG, LECT2 and SELENOP. These hepatokines contribute to NAFLD and NASH through interaction with some important genes PPARGC1A, FASN, NR1H4, SREBF1, PPARA, PPARG, TNF, INS, IL-18, ACSL4, EGFR and LEP.

## Hepatokines and adipogenesis signaling pathway

The adipose tissue is an important place for the human body to store adipose tissue, lipid metabolism, glucose sensitivity, and IR. It is also a key organization in metabolic diseases. Adipogenesis is the process of differentiation from preadipocytes into adipocytes and is coordinated by various transcription factors such as PPARγ and CCAAT/enhancer binding protein-α (C/EBPα) (48).

FGF21 inhibited the production of osteoblasts and stimulated the adipogenesis of bone marrow mesenchymal stem cells by enhancing the activity of PPAR-γ. Knockdown of FGF21 using a specific siRNA promotes the expression of PPAR-γ and C/EBPα but inhibits the expression of lipoprotein lipase (LPL) and preadipocyte factor 1 (Pref1). Furthermore, inhibition of nuclear factor erythroid related factor 2 (NRF2) attenuates adipogenesis and reduces the expression of FGF21 by mediating PPAR-γ in 3T3-L1 cells (48). Long-acting FGF21 partially affects the FGF21-adiponectin-IL17A pathway to reduce liver steatosis and inflammation in the NASH mouse model (49). These results indicate that FGF21 has a positive effect on adipogenesis. X-box binding protein 1 (XBP1) is an important transcription factor of the unfolded protein response. SIRT1 is an NAD+-dependent deacetylase that regulates diverse cellular functions largely *via* its ability to deacetylate key regulatory proteins to affect gene expression (50). FGF21 affects adipogenesis signaling by interacting with genes, such as XBP1, SIRT1, PPARG and SREBF1 (**Figure 1A**).

Fatty acid binding protein 4 (FABP4) is a new type of adipokine secreted by adipocytes that is related to lipolysis, and elevated serum FABP4 levels are related to obesity and IR (51). Knockdown of DPP-4 can reduce the gene expression and long-term secretion of FABP4 in 3T3-L1 adipocytes, and inhibition of DPP4 with sitagliptin can also reduce the expression and secretion of FABP4 in adipocytes (51). PPARγ agonists induce FABP4 with increased differentiation and fat accumulation and DPP4 knockdown promotes adipocyte maturation by mimicking growth factor withdrawal (an early step in adipocyte differentiation) (52). Another DPP-4 inhibitor, anagliptin, also could reduce the serum FABP4 concentration. SREBP-1 transcription factor regulates energy homeostasis by promoting adipogenesis, glycolysis and adipogenesis. FABP4 encodes the fatty acid binding protein found in adipocytes. The glucose transporter protein (SLC2A4 or GLUT4) is responsible for insulin-mediated glucose uptake in adipose tissue and skeletal muscle. DPP4 can affect adipogenesis signals by regulating TNF, SREBF-1, FABP4 and SLC2A4 (**Figure 2A**).

RBP4 and its membrane receptor STRA6 control adipogenesis by regulating cellular retinoid homeostasis and RARα activity (53). RBP4 may reduce obesity by regulating dietary vitamin A (retinol) to mediate the different effects of fat production in visceral fat compared with subcutaneous fat. Some findings indicate that RBP4 prevents adipogenesis by inhibiting the insulin pathway in humans. Furthermore, fenretinide inhibited the downregulation of PPARγ and improved insulin sensitivity and the levels of adiponectin, resistin, and serum RBP4 in adipose tissue (54). Activation of TLR4 signaling and the subsequent release of inflammatory cytokines has been postulated to be a critical player in connecting obesity-associated inflammation and insulin resistance. RBP4 interacts with TLR4, TNF, SLC2A4, PPARG, adipokine, and PPARA to regulate adipogenesis signaling (**Figure 2B**).

SERPINF1 gene participates in lipid metabolism and metabolic syndrome. Reduced SERPINF1 expression promotes adipogenesis and differentiation through upregulation of CD36. SERPINF1 negatively regulates adipogenesis by regulating various signaling intermediates (55) and blocks adipogenesis by inhibiting PPARγ, adiponectin and other adipocyte markers (56). Chen et al. found that SERPINF1 reduced lipid accumulation and fibrosis in liver and the differentiation of 3T3-L1 preadipocytes in *in vitro* assays, and SERPINF1 promotes lipolysis and prolongs cell cycle process through mTOR-S6K pathway and downstream transcription factors, such as PPARγ, CEBP-α and CEBP-β (57). These results indicate that SERPINF has different effects on adipogenesis through different signaling pathways. SERPINF1 could regulate adipogenesis signaling by interacting with TNF and PPARG (**Figure 3A**).

Plasma SHBG has clinical effects on lipid-mediated hepatic diseases and is considered a marker of NAFLD and metabolic disorders (58, 59). Exogenous SHBG treatment reduces PPARγ mRNA and protein levels (60). SHBG regulates adipogenesis signaling by interacting with TNF and PPARG (**Figure 4B**).

Adipose tissue (both subcutaneous and visceral) is a fundamental player in systemic inflammatory processes associated with obesity. Evidence for a key role of adipose tissue in NAFLD is accumulating. Adipose tissue with its enormous content of mediators communicates with metabolically active organs beyond the liver (61). ADIPOQ interacts with hepatokine FGF21, AHSG, SERPINF1, SHBG and FST (**Table 2**). LEP interacts indirectly with hepatokines such as SELENOP and FGF21 (**Table 2**). Meanwhile, LEP is a hub of adipokine network (**Figure 5D**). Resistin (RETN) is known as an adipose tissue-specific secretory factor and a cysteine-rich peptide hormone (derived from adipose tissue). RETN interacts directly with HMGB1 (**Table 2**). The negative effects of adipokines on NAFLD or NASH include: 1), activation of hepatic stellate cells (ADIPOQ, LEP and RETN); 2), recruitment of macrophages (ADIPOQ and NAMPT); and 3), activation of monocytes (ADIPOQ and RARRES2).

## Hepatokines and hepatic stellate cell activation/hepatic fibrosis signaling

The activation of HSC is one of liver non-parenchymal cells, which has been recognized as the main driver of fibrosis in human liver injury. As we all known, NAFLD is a multi-system disease, including liver fibrosis. The activation of HSC and hepatic fibrosis signaling will be an important pathogenic factor of NAFLD. Hepatokines can regulate HSC signaling and hepatic fibrogenesis by interacting with certain genes.

FGFs is involved in the development of the body. Hepatocytes and HSC produce several FGFs, and various FGFs have also been proven to directly induce HSC proliferation and activation, thereby realizing autocrine and paracrine regulation of HSC functions. Barb et al. found that circulating FGF21 levels in patients with NAFLD/NASH are elevated, and plasma FGF21 is correlated with the severity of steatohepatitis in NASH patients, especially fibrosis (62). In addition, studies have also reported that FGF21 has a correlation in lipid metabolism and fibrosis. C-X-C motif chemokine receptor 4 (CXCR4) is a chemokine receptor that plays a significant role in cellular functions, immune processes, growth and development, and liver disease. Chemokine (C-C motif) ligand 5 (CCL5) is a chemoattractant for monocytes, NK cells, T cells and eosinophils, and plays important roles in mediating inflammatory and immune responses in the liver. FGF21 could prevent the potential fibrosis in hepatic encephalopathy by inhibiting CXCR4/CCL5 activation and by up-regulating the production of IL-10 in the injured liver and mediating the STAT3-SOCS3 pathway to stimulate the production of pro-inflammatory cytokines and apoptosis of HSC. FGF21 attenuates hepatic fibrogenesis and inhibits the activation of HSC through TGF-β, NF-κB nuclear translocation, phosphorylation of Smad2/3 and IκBα (63). Furthermore, Xu et al. (64) indicated that FGF21 significantly attenuates platelet-derived growth factor-BB (PDGF-BB), induces HSC proliferation, prevents ECM production, and thereby alleviating hepatic fibrosis. FGF21 regulates hepatic fibrosis signaling by interacting with LEP and PPARG (**Figure 1A**).

Activation of ERK/c-JNK-MAPK and inhibition of the mTOR/STAT3 signaling pathway reveal the dose- and time-dependent role of HMGB1 in enhancing LX-2 (human HSC cell line) autophagy and fibrosis (65). Long noncoding Xist RNA (long noncoding RNA X-inactive specific transcript) enhances ethanol-induced autophagy and activation of HSCs through the miR-29b/HMGB1 axis (66). In NAFLD patients and mouse models, liver fibrosis can be treated by the mineralocorticoid receptor (MR)/osteopontin (OPN)/HMGB1 axis (67). HMGB1 mediates p65/miR-146b signaling and inhibits HNF1A to regulate hepatic fibrosis (68). Increasing levels of CC-chemokine ligand (CCL2) in NAFLD. Toll/interleukin-1 receptor-like protein (TIR) signaling and inflammatory cytokines regulate NF-κB activation. IL-17A associates with hepatic inflammation and damage. HMGB1 could regulate hepatic fibrosis signaling by interacting with TLR4, CCL2, TNF, TIR, IL17A, IL18 and INS (**Figure 2A**). These data enrich the functions of HMBG1 and its mechanism.

DPP4 inhibitor (DPP4-I) such as gemigliptin, vildagliptin, anagliptin, saxagliptin, sitagliptin and retelliptine, are drugs used clinically to lower blood glucose. DPP4-I can inhibit HSCs proliferation and expression of fibrosis related genes by inhibiting the phosphorylation of ERK1/2, p38 and Smad2/3, respectively (69). Anagliptin (DPP4-I) can inhibit HSC proliferation and liver fibrosis development, possibly through activation by Takeda G protein-coupled receptor 5 (TGR5) (70). CCL4-induced liver fibrosis can be alleviated by vildagliptin by targeting ERK1/2, p38α and NF-κB signals (71). DPP4 promotes fibrosis through energy metabolism, B cells, NK cells and CD4+ cells (72). In addition, DPP4 is involved in HSC signaling by interacting with TNF, EGFR, TNF and IL17A (**Figure 2A**).

Serum RBP4 could be linked to HSC by transporting vitamin A. There is a negative correlation between the level of plasma RBP4 and the order of severity of histological fibrosis, activity, and steatosis, which may be related to vitamin A. RBP4 can diagnose advanced fibrosis grades in patients with underlying chronic hepatitis C (73). RBP4 regulates HSC signaling by interacting with TNF, TLR4, AGT, PPARG and adipokines (**Figure 2B**).

Earlier *in vivo* and *in vitro* results showed that FST can inhibit HSC proliferation and hepatic fibrosis. FST can activate the liver progenitor cells (LPCs) signaling to control the expression of the controlled activin-HNF4α-coagulation factor axis to predict the occurrence and outcome of chronic liver failure (74). Follistatin-like 1 (FSTL1) is considered a proinflammatory mediator in various fibrosis-related and inflammatory diseases. It has been shown that knocking out Fstl1 will inhibit the TGF-β1/Smad3 signaling pathway, which will attenuate the activation of HSCs. A data suggests that TGF-β1-miR29a-Fstl1 regulatory circuit plays a key role in regulation the HSC activation and ECM production (75). FST could affect hepatic fibrosis signaling by interacting with TNF, PPARG, cytokines and INS (**Figure 3A**).

ASHG (a TGF-β antagonist) inhibits HSC activation, proliferation, and differentiation into myofibroblasts and protects against hepatic fibrogenesis by regulating TGF-β/Smad. ASHG interacts with TNF, TLR4, EGFR and INS to affect HSC activation and signaling pathways (**Figure 3B**). SERPINF1 prevents liver fibrosis and hepatic stellate cell activation by down-regulating the expression of PDGF receptor-α/β and blocking the phosphorylation of Akt and ERK induced by PDGF (76), which plays an important role in hepatic stellate cell activation. The two mechanisms by which SERPINF1 may inhibit liver cirrhosis are the direct inactivation of HSCs and the induction of apoptosis by activated HSCs. These facts suggest that SERPINF1 may be a marker of hepatic fibrosis. SERPINF1 is involved in hepatic fibrosis signaling by interacting with genes TNF and PPARG (**Figure 4A**).

High SHBG and low bioactive testosterone are related to liver fibrosis. In male patients with HCV-related chronic liver disease, low circulating free testosterone levels may be considered a risk factor for more advanced liver fibrosis, steatosis, and/or higher IR (77). SHBG is associated with HSC signaling by interacting with TNF and cytokines, and PPARG (**Figure 4B**). SELENOP levels are related to changes in metabolic characteristics and the degree of liver fibrosis (78). SELENOP is related to hepatic fibrosis signaling by interacting with TNF, EGFR and LEP (**Figure 5A**).

Hepatokines secreted by the liver have significant impact on the development of NAFLD and NASH into HCC (79). HSCs secrete stellakines such as APP, CCL2, CCL11, CSF1, CTGF, CXCL1, CXCL10, CXCL12, CXCL14, CXCL16, GAS6, NTN1, POSTN and WNT4 (80). APP is considered a hub of stellakine network (**Figure 5E**).

## Hepatokines and AMPK/NF-κB signaling

The main function of AMPK is to regulate intracellular energy metabolism and is considered a potential target to treat and prevent NAFLD. NF-κB family is composed of five cellular DNA binding subunits: p50, p52, cRel, p65 (also known as RelA), and RelB, encoded by NF-κB1, NF-κB2, REL, RELA, and RELB, respectively. The heterodimer, p50/p65, is the most common form of NF-ĸB and a key driver in liver cancer (81). NF-κB is an important inflammatory mediator, involved in the inflammatory pathway and lipid metabolism.

FGF21 directly stimulates the secretion of adiponectin and corticosteroids through FGFR1/β-klotho signaling or indirectly activates AMPK signaling to control metabolic disorders and the aging process (82). AKT2 has been shown to be induced in HCC and cholangiocarcinoma (83). ACC reduces hepatic fat content and markers of liver injury in patients with NASH. Carnitine palmitoyl transferase I (CPTI) is the key enzyme in the carnitine-dependent transport across the mitochondrial inner membrane. FGF21 exerts a preventive effect by activating AMPK-AKT2-NRF2 and AMPK-ACC-CPT1 (84). FGF21 improves rheumatoid arthritis through antioxidant response and inhibition of NF-κB inflammation pathway (85). Human FGF21 mediates the NF-κB-mediated TGF-β signaling pathway, which leads to the suppression of inflammation. Some scholars have added this mechanism by which FGF21 can inhibit inflammation and apoptosis because it could inhibit the TLR4/myeloid differentiation 88 (MYD88)/NF-κB signaling pathway (86). Inhibiting NF-κB/NOD-like receptor family pyrin domain containing 3 (NLRP3) inflammasome activation can activate the protective effect of FGF21 (87). FGF 21 inhibits HSC activation and attenuates liver fibrosis through NF-κB signaling pathways (63). FGF-21 can inhibit the activation of stress kinase and NF-κB, which will prevent palmitate-induced IR in human skeletal muscle myotubes (88). FGF21 regulates the polarization of macrophages by mediating AMPK/NF-κB signaling pathway to reduce liver aging damage (89). FGF21 is involved in AMPK or NF-κB signaling by interacting with FASN, Acetyl-CoA carboxylase alpha (ACACA), carnitine palmitoyl-transferase 1A (CPT1A), LIPE, ADIPOQ, SIRT1, PPARGC1A and glucose transporter protein 1 (SLC2A1 or GLUT1), casein kinase 2 (CK2), and TNF (**Figure 1A**).

In animal and cell IR models, DPP4 expression is inhibited by AMPK activation by JAK2/STAT3 signaling in adipocytes (90). It was subsequently reported that DPP4 inhibition or silencing can improve endothelial senescence by regulating AMPK/SIRT1/NRF2 *in vivo* and *in vitro* (91). Vildagliptin modulates ERK1/2, p38α and NF-κB signaling to reduce CCL4-induced liver fibrosis (71). Saxagliptin improves oxidized low-density lipoprotein cholesterol (ox-LDL)-induced endothelial dysfunction by regulating suppressed activator protein 1 (AP-1) and NF-κBp65 accumulation and inhibiting its promoter activity (92). Sitagliptin also contributes to protecting NAFLD by downregulating the HMGB1/TLR4/NF-κB signaling pathway. DPP4 is involved in p38 AMPK signaling by interacting with TNF and mitogen-activated protein kinase 5 (MAP3K5) (**Figure 2A**).

In an atherosclerosis study, RBP4 is positively correlated with NF-κB, and RBP4 may participate in the inflammatory pathway by regulating NF-κB signaling. RBP4 mediates NADPH oxidase and NF-κB-dependent and retinol-independent mechanisms to induce inflammation in human endothelial cells (93). Lipase E (LIPE) is expressed in adipose tissues and mobilizes fats for energy. RBP4 is associated with AMP signaling by interacting with FASN, LIPE, SLC2A4, PPARGC1A and adipokines (**Figure 2B**).

FST promotes WAT browning, possibly by activating AMPK- PGC1α-fibronectin type III domain-containing protein 5 (Fndc5) and pp38MAPK/pERK1/2 pathways, thereby activating the insulin pathway to promote metabolism. FSTL1 can inhibit AMPK phosphorylation, and AMPK inhibitors can reverse the antiproliferative effect of FSTL1 on the blood vessel wall (94). However, FSTL1 stimulates glucose uptake (95) by activating AMPK signaling. Some findings show that FSTL1 accelerates the progression of rheumatoid arthritis by activating the MAPK, JAK/STAT3 and NF-κB pathways to enhance TLR4 and promote the secretion of matrix metalloproteinase (96). FST can interact with INS, SLC2A4, ADIPOQ and PPARGC1A to regulate AMPK signaling pathways (**Figure 3A**).

Studies have found that rosiglitazone inhibits the expression and secretion of SERPINF1 in fat and liver by promoting AMPK phosphorylation, which is closely related to the improvement of IR (97). Furthermore, ubiquitin-dependent proteasome SERPINF1/PEDFR/PPARγ axis degrades AMPKα and reduces the formation of ATP (98). The interaction of SERPINF1 and genes (ADIPOQ and PPARGC1AA) affect AMPK signaling pathway (**Figure 4A**).

Adiponectin activates AMPK to promote SHBG production, thereby reducing liver lipid content and increasing HNF4α levels. AMPK/PPARγ interaction regulates HNF4α to affect the expression of SHBG, which is a prerequisite for SHBG upregulation (99). TNF-α plays an important role in downregulating SHBG through JNK and NF-kB pathways in NAFLD (100). The interaction of SHBG with genes (ADIPOQ and FASN) affect the AMPK signaling pathway (**Figure 4B**)

Gemigliptin (a novel DPP4 inhibitor) might mitigate hepatic steatosis and IR, suggesting a direct protective effect on NAFLD progression, which inhibits the expression of LECT2 through AMPK-dependent and JNK-dependent mechanisms (36). In addition, research has shown that AMPK negatively regulates LECT2 (101). LECT2 affects NF-κB signaling through its interaction with TNF and cytokines (**Figure 4C**).

LDL receptor-related protein-1 (LRP1) is a ubiquitous receptor with both cell signaling and ligand endocytosis properties. Misu et al. also found (101) that SELENOP reduces the expression mRNA level of PPARC1A (genes expressed downstream of AMPK) in the presence of H2O2 and SELENOP inhibits the reactive oxygen species (ROS)/AMPK/PGC-1α pathway in skeletal muscle through its receptor LRP1. In addition, the authors showed *in vitro* that *de novo* lipogenesis and increased fatty acid oxidation can be regulated by SELENOP through AMPK/ACC pathway mediated triglyceride overload in hepatocytes. SELENOP also can be elevated in NAFLD and participates in NAFLD pathogenesis through AMPK/ACC pathway. In addition, the authors showed *in vitro* that SELENOP can regulate *de novo* lipogenesis and increase fatty acid oxidation (FAO) through AMPK/ACC pathway mediated triglyceride overload in hepatocytes. SELENOP also can be elevated in NAFLD and participates in NAFLD pathogenesis through AMPK/ACC pathway (102). TNF, EGFR and SELENOPP are associated with NF-κB signaling (**Figure 5A**). Curcumin may reduce the cytoplasmic and nuclear translocation of HMGB1-NF-κB, which reduces oxidative stress and blood glucose levels in the liver of NASH patients (103).

Based on the pathogenesis of NAFLD, it could be found that NF-κB and AMPK signaling pathways are closely related with the incidence of NAFLD. Interplay of FGF21 and the inflammatory cytokine TNF can activate p38/AMPK and NF-κB signaling, and in turn, the activation of these pathways could increase the expression of inflammatory cytokines. Interaction of FGF21/SHBG/RBP4 and adiponectin could reduce fatty acid synthesis and enhance FAO *via* activation of AMPK, and then resist steatosis and increase insulin sensitivity, but also could activate PPARα and inhibit NF-kB pathway. Leptin, a key player of adipokine network, may also stimulate AMPK to increase in β-oxidation.

## Hepatokines and Type2 diabetes mellitus signaling

T2DM is a type of multisystem complex disease that influences human life, health, and safety. It can also cause many complications, such as NAFLD and NASH. The important pathogenic factors of T2DM include genetic factors, environmental factors, poor lifestyles, IR, pancreatic β-cell dysfunction, glucagon, etc.

FGF21 has insulin sensitivity and induces glucose uptake to reduce glucose concentration. Another mechanism for FGF21 to regulate insulin sensitivity is to send signals directly to adipose tissue (104, 105). In mouse experimental models, it was found that the activation of FGF21 induced by glycolipid toxicity can mediate islet autophagy by inhibiting the phosphorylation of AMPK and stimulating the expression of LC3 (autophagy marker) (106). Furthermore, Liraglutide induces the expression of FGF21 in macrophages, and then activates the liver kinase B1(LKB1)-AMPK- ACC1 pathway in an autocrine manner to regulate the lipid metabolism in white adipose tissue and macrophages in T2DM mice (107). However, researchers also found that pancreatic FGF21 promotes the expression of major insulin secretion protein, such as insulin gene transcription factor and soluble N-ethylmaleimide-sensitive factor attachment protein receptor (SNARE) protein, and activates phosphatidylinositol 3-kinase (PI3K)/Akt signaling-dependent insulin expression and secretion to protect T2DM in mice (108). Although FGF21 has been found in mouse studies to directly or indirectly affect T2DM, the specific mechanism by which FGF21 regulatesT2DM signaling in humans is scarce. In general, FGF21 has good therapeutic potential in T2DM and NAFLD. FGF21 can mediate T2DM signaling to regulate body metabolism through interaction with TNF, ADIPOQ, PPARG, SLC2A4 and INS (**Figure 1A**).

Some researchers found that circulating and plasma FST levels associated with glycemic parameters are highly elevated in patients with T2DM (109–111). Park K, et al. found that patients with T2DM have lower circulating levels of FSLT1. However, Xu et al. demonstrated that demonstrated that serum FSTL1 levels are increased in newly diagnosed patients with T2DM, which are associated with glucose metabolism and IR, the secretion and release of FSTL1 were regulated by hyperinsulinemia, FFA, and physical activity (112). There are also reports that FSTL1 can mediate AMPK pathway to stimulate myocardial oxygen consumption and glucose uptake (113, 114). Furthermore, FST can increase the expression of UCP1. FST interacts with TNF, PPARG, TNS and cytokines in regulating T2DM (**Figure 3A**).

ASHG was shown to be an inhibitor of IRS1, which leads to IR. It means that ASHG might play a crucial role in regulation of insulin sensitivity. ASHG through inhibits insulin receptor tyrosine resulting in IR (115). ASHG throughTLR4 promote lipid-induced IR, which may be a new therapeutic target for managing IR and T2DM (116). Furthermore, high glucose levels increase the expression of ASHG by activating the ERK-1–ERK-2 signaling pathway. The above facts indicate that ASHG may be an important way to activate T2DM signaling through mediating glucose metabolism and IR. Moreover, ASHG also could interact with TNF, ADIPOQ, SLC2A4 and INS to regulate T2DM signaling (**Figure 3B**).

SERPINF1 plays an important role in diabetes metabolism. In children with T2DM, SERPINF1 has a positive association with lean mass, fat mass, and insulin (117). Insulin can downregulate SERPINF1 expression and increase glucose uptake in T2DM adipocytes, which may be one of the mechanisms to improve peripheral IR. SERPINF1 can affect T2DM signaling by interacting with ACSL4, TNF, Ck2, ADIPOQ and PPARG (**Figure 4A**).

SHBG has a significant correlation with T2DM. SHBG is a marker of IR and can be used to identify individuals with IR for targeted therapy with insulin sensitizers. SHBG interacts with TNF, ADIPOQ and PPARG to regulate T2DM signaling (**Figure 4B**).

LECT2 is a protein secreted by the liver that regulates energy metabolism and contributes to T2DM. Recent studies found that serum levels of LECT2 positively correlate with measures of obesity, the severity of liver steatosis and IR in both mouse models and humans (35). Lan et al. found that there is a positive correlation between circulating LECT2 levels and human IR, lack of FGF21 in mice insulin sensitivity of skeletal muscle is improved, and administration of recombinant LECT2 in mice can lead to impaired insulin signaling and induce skeletal muscle IR (35). They also found that LECT2 impairs insulin signaling by activating JNK in C2C12 myotubes, and lack of LECT2 in mice mediates activation of Akt phosphorylation to improve insulin sensitivity in skeletal muscle (35). LECT2 can induce mTOR phosphorylation, SREBP-1 cleavage, and lipid accumulation in hepatocytes through a JNK-dependent mechanism (36). In addition to these, LECT2 can also regulate T2DM signaling through its interaction with TNF and PPARG (**Figure 4C**).

Several cytokines or peptides are Adipose tissue-derived adipokines (**Figure 5D**), HSC-derived stellakines (**Figure 5E**), muscle-derived myokines (**Figure 5F**), and hepatocyte-derived hepatokines participate in response to certain nutrition and/or physical activity conditions. Contracting skeletal muscle generates and releases a variety of cytokines and other peptides, which are collectively termed “myokines”. Myokine mediates muscle myogenesis and regeneration, and communicates with liver, adipose tissue, and pancreas (**Table 3**). Physical inactivity can change the production profile of myokines and their responses since the production of most myokines is affected by muscle contraction. The action of myokines for exercise-induced adaptation in skeletal muscle is responsible for oxidation and lipolysis of fatty acid and disposal of glucose (118).

## Conclusion

The beneficial effects of hepatokines include 1) improvement of insulin sensitivity (FGF21); 2) prevention of liver fibrosis (FGF21, HMGB1, PST, AHSG and SERPINF1) and 3) reduction of inflammation and lipid accumulation (FGF21, HMGB1 and LECT2). Hepatokines are involved in NAFLD or NASH pathogenesis: 1) promotion of inflammation, IR, and fibrosis (DPP4) and 2) progression from simple steatosis to NASH (PST and LECT2). Future directions in this field include: 1) determining how the interaction between hepatokines and other genes regulate NAFLD and NASH *via* interorgan and cell-cell crosstalk; 2) dissecting how preclinical evidence for hepatokines translates to these diseases; 3) understanding how hepatokines contribute to NAFLD and NASH progression; and 4) recognizing the interaction of each hepatokine and other genes under healthy and pathophysiological conditions.

At present, the therapeutic potential of hepatokines, adipokines and inflammatory mediators has not been effectively confirmed in clinical practice (119), and more research is needed to explore the mystery between them. These multiple regulations in **Table 2** between hepatokines and adipokines could lead to potential effective treatments to interfere with the development of NAFLD and NASH. Hepatokines such as DPP4, FST, AHSG, HMGB1 and RBP4 can regulate IL-6 directly or indirectly. IL-15 and FST regulate DPP4 and MSTN **(** **Table 3**), respectively, suggesting that the interaction between hepatokines and myokines could provide some novel trail to prevent and treat T2DM.

As shown in **Table 4**, interplays between stellakines and hepatokines include the following: 1) HMGB1 interacts with APP, CCL2, CXCL1, CXCL10 and CXCL12; 2), SERPINF1 interacts with APP and CCN2; 3) DPP4 interacts with CCL11, CXCL10 and CXCL12; and 4), CXCL10 interacts with LECT2. In view of the irreplaceable role of HSCs in NAFLD and NASH, interplays of HSCs-derived stellakines and hepatokines may be very helpful for finding new treatments for these diseases.

**Table 4:** IPA analysis of Cell-cell signaling and interaction of muscle-derived myokine.

Myokine	Cell-cell signaling and function	Interaction between myokine and hepaokines
**IL-6**	Activation of hepatic stellate cells; Binding of hepatocytes; Sensitization of macrophage; Proinflammatory cytokine associated with insulin resistance in obesity.	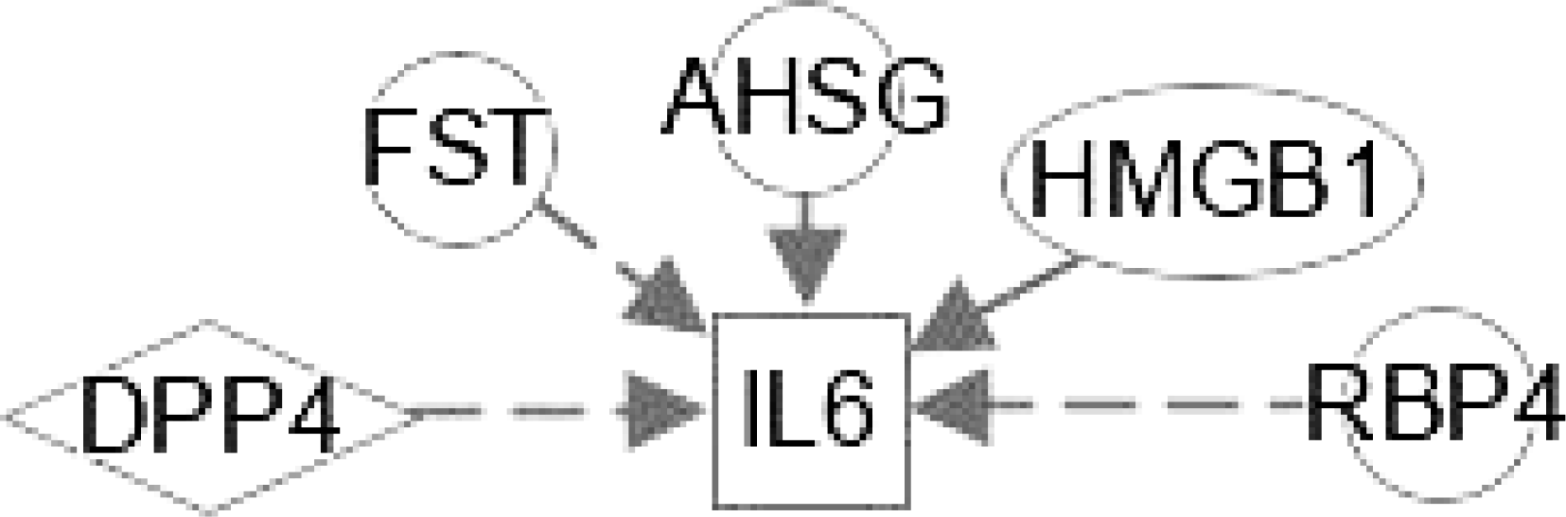
**MSTN**	Insulin sensitivity; Activation of stellate cell; non-insulin-dependent diabetes mellitus; Chemotaxis of macrophages; Accumulation of fat; synthesis and concentration of triacylglycerol.	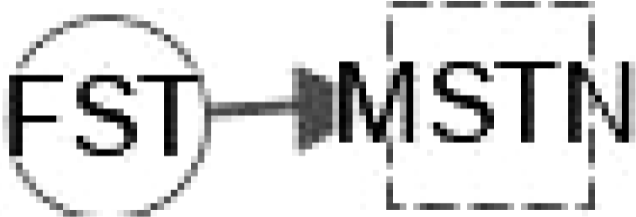
**FNDC5**	Darkening of white adipose tissue; Improvement of insulin sensitivity and induction of weight loss; Increase of energy expenditure.	Undetected
**AGXT2**	Reduction of insulin resistance; Synthesis of glycine; Browning of adipose tissue; lipid oxidation.	Undetected
**IL-15**	Deposition of lipid; Oxidation of fat; Suppression of macrophages; Adhesion and stimulation of monocytes; Release after acute episodes of aerobic exercise; Anti-inflammatory properties by inhibiting TNF-α expression.	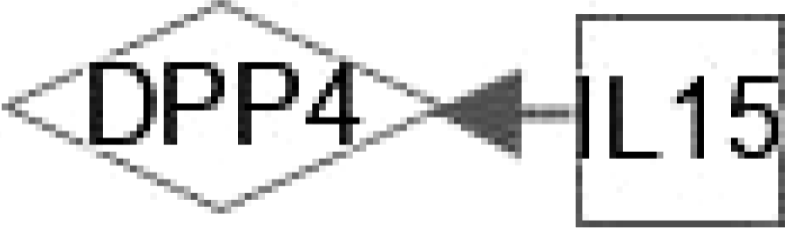
**FST**	Impairment of glucose tolerance; Inhibition of the actions of myostatin; contribution to hypertrophy of skeletal muscle and reduction in fat mass; Expression in the context of physical activity, especially aerobic; Resistance or high intensity training.	Undetected
**ERFE**	Autophagy of liver cells; Increase of the uptake of lipids by adipose tissue and liver; Decrease of the plasma concentration of free fatty acid.	Undetected
**SPARC**	Activation of hepatic stellate cells; Binding of endothelial cells; Resistance exercises and muscle hypertrophy; Inhibition of adipose tissue formation; Increase of insulin release and optimization of glucose uptake.	Undetected
**METRNL**	Activation of macrophages; Inflammation of liver.	Undetected
**BDNF**	Insulin resistance type A; Muscle and brain induction after exercise; Increase of sensitivity to insulin.	Undetected
**FGF21**	Insulin resistance; Binding of hepatocytes; Responsiveness of brown adipocytes; Hepatic steatosis; Impaied glucose tolerance.	Undetected

Ingenuity pathway analysis (IPA) of myokines. All identified disease and function of myokines were screened individually. Representative cell-cell signaling and interaction of myokines are associated with disease and function. IL-6, Interleukin 6; MSTN, Myostatin; AGXT2, Alanine--glyoxylate aminotransferase 2; IL-15, Interleukin -15; FST, Follistatin; ERFE, Myonectin; SPARC, Secreted Protein Acidic and Rich in Cysteine; METRNL, Meteorin Like; BDNF, Brain derived neurotrophic factor.

The signaling of FXR/RXR, hepatic fibrosis, AMPK/NF-κB, T2DM and adipogenesis are interconnected to a regulatory network, and blocking any one of these pathways cannot be effective for the prevention and treatment of NAFLD or NASH. Therefore, a more in-depth study of liver cell-cell signaling, and interaction is needed.

## Author contributions

BY, LL, and DZ contributed equally to reviewing the literature and drafting the manuscript. WF assisted in the literature review and drafting the manuscript. LB-T and JS provided critical reading of the manuscript. HY and XY provided critical editing and revisions of the manuscript. All authors contributed to the article and approved the submitted version.

## Funding

This work was supported by Medical and Health Appropriate Technology Development, Promotion and Application Project of Guangxi, S201664, NIH grants R01CA172086 (HP Yang, JM Mato, and SC Lu) and P01CA233452 (HP Yang, E Seki, and SC Lu). Graduate Innovation projects of Central South University (1053320182802 to L. L). The funders had no role in study design, data collection and analysis, decision to publish, or preparation of the manuscript.

## Conflict of interest

The authors declare that the research was conducted in the absence of any commercial or financial relationships that could be construed as a potential conflict of interest.

## Publisher’s note

All claims expressed in this article are solely those of the authors and do not necessarily represent those of their affiliated organizations, or those of the publisher, the editors and the reviewers. Any product that may be evaluated in this article, or claim that may be made by its manufacturer, is not guaranteed or endorsed by the publisher.
