# Corrigendum: Regulatory network and interplay of hepatokines, stellakines, myokines and adipokines in nonalcoholic fatty liver diseases and nonalcoholic steatohepatitis

**DOI:** 10.3389/fendo.2023.1284809

**Published:** 2023-09-28

**Authors:** Bing Yang, Liqing Lu, Dongmei Zhou, Wei Fan, Lucía Barbier-Torres, Justin Steggerda, Heping Yang, Xi Yang

**Affiliations:** ^1^ Department of Geriatric Endocrinology and Metabolism, Guangxi Key Laboratory of Precision Medicine in Cardio-cerebrovascular Diseases Control and Prevention, Guangxi Clinical Research Center for Cardio-cerebrovascular Diseases, The First Affiliated Hospital of Guangxi Medical University, Nanning, China; ^2^ Department of Thoracic Surgery, Xiangya Hospital, Central South University, Changsha, China; ^3^ Division of Digestive and Liver Diseases, Cedars-Sinai Medical Center, Los Angeles, CA, United States; ^4^ Department of Surgery, Cedars-Sinai Medical Center, Los Angeles, CA, United States

**Keywords:** hepatokines, stellakines, myokines, adipokines, nonalcoholic fatty liver disease, nonalcoholic steatohepatitis

In the published article, there was an error. Paragraphs 9-16 were a duplicate of Paragraphs 1-8 in section **Hepatokines and Type2 diabetes mellitus signaling**. This section previously stated:

“T2DM is a type of multisystem complex disease that influences human life, health, and safety. It can also cause many complications, such as NAFLD and NASH. The important pathogenic factors of T2DM include genetic factors, environmental factors, poor lifestyles, IR, pancreatic b-cell dysfunction, glucagon, etc.

FGF21 has insulin sensitivity and induces glucose uptake to reduce glucose concentration. Another mechanism for FGF21 to regulate insulin sensitivity is to send signals directly to adipose tissue (104, 105). In mouse experimental models, it was found that the activation of FGF21 induced by glycolipid toxicity can mediate islet autophagy by inhibiting the phosphorylation of AMPK and stimulating the expression of LC3 (autophagy marker) (106). Furthermore, Liraglutide induces the expression of FGF21 in macrophages, and then activates the liver kinase B1(LKB1)- AMPK- ACC1 pathway in an autocrine manner to regulate the lipid metabolism in white adipose tissue and macrophages in T2DM mice (107). However, researchers also found that pancreatic FGF21 promotes the expression of major insulin secretion protein, such as insulin gene transcription factor and soluble N-ethylmaleimide-sensitive factor attachment protein receptor (SNARE) protein, and activates phosphatidylinositol 3- kinase (PI3K)/Akt signaling-dependent insulin expression and secretion to protect T2DM in mice (108). Although FGF21 has been found in mouse studies to directly or indirectly affect T2DM, the specific mechanism by which FGF21 regulatesT2DM signaling in humans is scarce. In general, FGF21 has good therapeutic potential in T2DM and NAFLD. FGF21 can mediate T2DM signaling to regulate body metabolism through interaction with TNF, ADIPOQ, PPARG, SLC2A4 and INS (Figure 1).

Some researchers found that circulating and plasma FST levels associated with glycemic parameters are highly elevated in patients with T2DM (109–111). Park K, et al. found that patients with T2DM have lower circulating levels of FSLT1. However, Xu et al. demonstrated that demonstrated that serum FSTL1 levels are increased in newly diagnosed patients with T2DM, which are associated with glucose metabolism and IR, the secretion and release of FSTL1 were regulated by hyperinsulinemia, FFA, and physical activity (112). There are also reports that FSTL1 can mediate AMPK pathway to stimulate myocardial oxygen consumption and glucose uptake (113, 114). Furthermore, FST can increase the expression of UCP1. FST interacts with TNF, PPARG, TNS and cytokines in regulating T2DM (Figure 3A).

ASHG was shown to be an inhibitor of IRS1, which leads to IR. It means that ASHG might play a crucial role in regulation of insulin sensitivity. ASHG through inhibits insulin receptor tyrosine resulting in IR (115). ASHG throughTLR4 promote lipid-induced IR, which may be a new therapeutic target for managing IR and T2DM (116). Furthermore, high glucose levels increase the expression of ASHG by activating the ERK-1–ERK- 2 signaling pathway. The above facts indicate that ASHG may be an important way to activate T2DM signaling through mediating glucose metabolism and IR. Moreover, ASHG also could interact with TNF, ADIPOQ, SLC2A4 and INS to regulate T2DM signaling (Figure 3B).

SERPINF1 plays an important role in diabetes metabolism. In children with T2DM, SERPINF1 has a positive association with lean mass, fat mass, and insulin (117). Insulin can downregulate SERPINF1 expression and increase glucose uptake in T2DM adipocytes, which may be one of the mechanisms to improve peripheral IR. SERPINF1 can affect T2DM signaling by interacting with ACSL4, TNF, Ck2, ADIPOQ and PPARG (Figure 4A).

SHBG has a significant correlation with T2DM. SHBG is a marker of IR and can be used to identify individuals with IR for targeted therapy with insulin sensitizers. SHBG interacts with TNF, ADIPOQ and PPARG to regulate T2DM signaling (Figure 4B).

LECT2 is a protein secreted by the liver that regulates energy metabolism and contributes to T2DM. Recent studies found that serum levels of LECT2 positively correlate with measures of obesity, the severity of liver steatosis and IR in both mouse models and humans (35). Lan et al. found that there is a positive correlation between circulating LECT2 levels and human IR, lack of FGF21 in mice insulin sensitivity of skeletal muscle is improved, and administration of recombinant LECT2 in mice can lead to impaired insulin signaling and induce skeletal muscle IR (35). They also found that LECT2 impairs insulin signaling by activating JNK in C2C12 myotubes, and lack of LECT2 in mice mediates activation of Akt phosphorylation to improve insulin sensitivity in skeletal muscle (35). LECT2 can induce mTOR phosphorylation, SREBP-1 cleavage, and lipid accumulation in hepatocytes through a JNK-dependent mechanism (36). In addition to these, LECT2 can also regulate T2DM signaling through its interaction with TNF and PPARG (Figure 4C).

Several cytokines or peptides are Adipose tissue-derived adipokines (Figure 5D), HSC-derived stellakines (Figure 5E), muscle-derived myokines (Figure 5F), and hepatocyte-derived hepatokines participate in response to certain nutrition and/or physical activity conditions. Contracting skeletal muscle generates and releases a variety of cytokines and other peptides, which are collectively termed “myokines”. Myokine mediates muscle myogenesis and regeneration, and communicates with liver, adipose tissue, and pancreas (Table 3). Physical inactivity can change the production profile of myokines and their responses since the production of most myokines is affected by muscle contraction. The action of myokines for exercise-induced adaptation in skeletal muscle is responsible for oxidation and lipolysis of fatty acid and disposal of glucose (118).

T2DM is a type of multisystem complex disease that influences human life, health, and safety. It can also cause many complications, such as NAFLD and NASH. The important pathogenic factors of T2DM include genetic factors, environmental factors, poor lifestyles, IR, pancreatic b-cell dysfunction, glucagon, etc.

FGF21 has insulin sensitivity and induces glucose uptake to reduce glucose concentration. Another mechanism for FGF21 to regulate insulin sensitivity is to send signals directly to adipose tissue (104, 105). In mouse experimental models, it was found that the activation of FGF21 induced by glycolipid toxicity can mediate islet autophagy by inhibiting the phosphorylation of AMPK and stimulating the expression of LC3 (autophagy marker) (106). Furthermore, Liraglutide induces the expression of FGF21 in macrophages, and then activates the liver kinase B1(LKB1)-AMPK- ACC1 pathway in an autocrine manner to regulate the lipid metabolism in white adipose tissue and macrophages in T2DM mice (107). However, researchers also found that pancreatic FGF21 promotes the expression of major insulin secretion protein, such as insulin gene transcription factor and soluble N-ethylmaleimide-sensitive factor attachment protein receptor (SNARE) protein, and activates phosphatidylinositol 3-kinase (PI3K)/Akt signaling- dependent insulin expression and secretion to protect T2DM in mice (108). Although FGF21 has been found in mouse studies to directly or indirectly affect T2DM, the specific mechanism by which FGF21 regulatesT2DM signaling in humans is scarce. In general, FGF21 has good therapeutic potential in T2DM and NAFLD. FGF21 can mediate T2DM signaling to regulate body metabolism through interaction with TNF, ADIPOQ, PPARG, SLC2A4 and INS (Figure 1A).

Some researchers found that circulating and plasma FST levels associated with glycemic parameters are highly elevated in patients with T2DM (109–111). Park K, et al. found that patients with T2DM have lower circulating levels of FSLT1. However, Xu et al. demonstrated that demonstrated that serum FSTL1 levels are increased in newly diagnosed patients with T2DM, which are associated with glucose metabolism and IR, the secretion and release of FSTL1 were regulated by hyperinsulinemia, FFA, and physical activity (112). There are also reports that FSTL1 can mediate AMPK pathway to stimulate myocardial oxygen consumption and glucose uptake (113, 114). Furthermore, FST can increase the expression of UCP1. FST interacts with TNF, PPARG, TNS and cytokines in regulating T2DM (Figure 3A).

ASHG was shown to be an inhibitor of IRS1, which leads to IR. It means that ASHG might play a crucial role in regulation of insulin sensitivity. ASHG through inhibits insulin receptor tyrosine resulting in IR (115). ASHG throughTLR4 promote lipid-induced IR, which may be a new therapeutic target for managing IR and T2DM (116). Furthermore, high glucose levels increase the expression of ASHG by activating the ERK-1–ERK- 2 signaling pathway. The above facts indicate that ASHG may be an important way to activate T2DM signaling through mediating glucose metabolism and IR. Moreover, ASHG also could interact with TNF, ADIPOQ, SLC2A4 and INS to regulate T2DM signaling (Figure 3B).

SERPINF1 plays an important role in diabetes metabolism. In children with T2DM, SERPINF1 has a positive association with lean mass, fat mass, and insulin (117). Insulin can downregulate SERPINF1 expression and increase glucose uptake in T2DM adipocytes, which may be one of the mechanisms to improve peripheral IR. SERPINF1 can affect T2DM signaling by interacting with ACSL4, TNF, Ck2, ADIPOQ and PPARG (Figure 4A).

SHBG has a significant correlation with T2DM. SHBG is a marker of IR and can be used to identify individuals with IR for targeted therapy with insulin sensitizers. SHBG interacts with TNF, ADIPOQ and PPARG to regulate T2DM signaling (Figure 4B).

LECT2 is a protein secreted by the liver that regulates energy metabolism and contributes to T2DM. Recent studies found that serum levels of LECT2 positively correlate with measures of obesity, the severity of liver steatosis and IR in both mouse models and humans (35). Lan et al. found that there is a positive correlation between circulating LECT2 levels and human IR, lack of FGF21 in mice insulin sensitivity of skeletal muscle is improved, and administration of recombinant LECT2 in mice can lead to impaired insulin signaling and induce skeletal muscle IR (35). They also found that LECT2 impairs insulin signaling by activating JNK in C2C12 myotubes, and lack of LECT2 in mice mediates activation of Akt phosphorylation to improve insulin sensitivity in skeletal muscle (35). LECT2 can induce mTOR phosphorylation, SREBP-1 cleavage, and lipid accumulation in hepatocytes through a JNK-dependent mechanism (36). In addition to these, LECT2 can also regulate T2DM signaling through its interaction with TNF and PPARG (Figure 4C).

Several cytokines or peptides are adipose tissue-derived adipokines (Figure 5D), HSC-derived stellakines (Figure 5E), muscle-derived myokines (Figure 5F), and hepatocyte-derived hepatokines participate in response to certain nutrition and/or physical activity conditions. Contracting skeletal muscle generates and releases a variety of cytokines and other peptides, which are collectively termed “myokines”. Myokine mediates muscle myogenesis and regeneration, and communicates with liver, adipose tissue, and pancreas (Table 3). Physical inactivity can change the production profile of myokines and their responses since the production of most myokines is affected by muscle contraction. The action of myokines for exercise-induced adaptation in skeletal muscle is responsible for oxidation and lipolysis of fatty acid and disposal of glucose (118).”

The corrected section appears below:

“T2DM is a type of multisystem complex disease that influences human life, health, and safety. It can also cause many complications, such as NAFLD and NASH. The important pathogenic factors of T2DM include genetic factors, environmental factors, poor lifestyles, IR, pancreatic b-cell dysfunction, glucagon, etc.

FGF21 has insulin sensitivity and induces glucose uptake to reduce glucose concentration. Another mechanism for FGF21 to regulate insulin sensitivity is to send signals directly to adipose tissue (104, 105). In mouse experimental models, it was found that the activation of FGF21 induced by glycolipid toxicity can mediate islet autophagy by inhibiting the phosphorylation of AMPK and stimulating the expression of LC3 (autophagy marker) (106). Furthermore, Liraglutide induces the expression of FGF21 in macrophages, and then activates the liver kinase B1(LKB1)- AMPK- ACC1 pathway in an autocrine manner to regulate the lipid metabolism in white adipose tissue and macrophages in T2DM mice (107). However, researchers also found that pancreatic FGF21 promotes the expression of major insulin secretion protein, such as insulin gene transcription factor and soluble N-ethylmaleimide-sensitive factor attachment protein receptor (SNARE) protein, and activates phosphatidylinositol 3- kinase (PI3K)/Akt signaling-dependent insulin expression and secretion to protect T2DM in mice (108). Although FGF21 has been found in mouse studies to directly or indirectly affect T2DM, the specific mechanism by which FGF21 regulatesT2DM signaling in humans is scarce. In general, FGF21 has good therapeutic potential in T2DM and NAFLD. FGF21 can mediate T2DM signaling to regulate body metabolism through interaction with TNF, ADIPOQ, PPARG, SLC2A4 and INS (Figure 1A).

Some researchers found that circulating and plasma FST levels associated with glycemic parameters are highly elevated in patients with T2DM (109–111). Park K, et al. found that patients with T2DM have lower circulating levels of FSLT1. However, Xu et al. demonstrated that demonstrated that serum FSTL1 levels are increased in newly diagnosed patients with T2DM, which are associated with glucose metabolism and IR, the secretion and release of FSTL1 were regulated by hyperinsulinemia, FFA, and physical activity (112). There are also reports that FSTL1 can mediate AMPK pathway to stimulate myocardial oxygen consumption and glucose uptake (113, 114). Furthermore, FST can increase the expression of UCP1. FST interacts with TNF, PPARG, TNS and cytokines in regulating T2DM (Figure 3A).

ASHG was shown to be an inhibitor of IRS1, which leads to IR. It means that ASHG might play a crucial role in regulation of insulin sensitivity. ASHG through inhibits insulin receptor tyrosine resulting in IR (115). ASHG throughTLR4 promote lipid-induced IR, which may be a new therapeutic target for managing IR and T2DM (116). Furthermore, high glucose levels increase the expression of ASHG by activating the ERK-1–ERK- 2 signaling pathway. The above facts indicate that ASHG may be an important way to activate T2DM signaling through mediating glucose metabolism and IR. Moreover, ASHG also could interact with TNF, ADIPOQ, SLC2A4 and INS to regulate T2DM signaling (Figure 3B).

SERPINF1 plays an important role in diabetes metabolism. In children with T2DM, SERPINF1 has a positive association with lean mass, fat mass, and insulin (117). Insulin can downregulate SERPINF1 expression and increase glucose uptake in T2DM adipocytes, which may be one of the mechanisms to improve peripheral IR. SERPINF1 can affect T2DM signaling by interacting with ACSL4, TNF, Ck2, ADIPOQ and PPARG (Figure 4A).

SHBG has a significant correlation with T2DM. SHBG is a marker of IR and can be used to identify individuals with IR for targeted therapy with insulin sensitizers. SHBG interacts with TNF, ADIPOQ and PPARG to regulate T2DM signaling (Figure 4B).

LECT2 is a protein secreted by the liver that regulates energy metabolism and contributes to T2DM. Recent studies found that serum levels of LECT2 positively correlate with measures of obesity, the severity of liver steatosis and IR in both mouse models and humans (35). Lan et al. found that there is a positive correlation between circulating LECT2 levels and human IR, lack of FGF21 in mice insulin sensitivity of skeletal muscle is improved, and administration of recombinant LECT2 in mice can lead to impaired insulin signaling and induce skeletal muscle IR (35). They also found that LECT2 impairs insulin signaling by activating JNK in C2C12 myotubes, and lack of LECT2 in mice mediates activation of Akt phosphorylation to improve insulin sensitivity in skeletal muscle (35). LECT2 can induce mTOR phosphorylation, SREBP-1 cleavage, and lipid accumulation in hepatocytes through a JNK-dependent mechanism (36). In addition to these, LECT2 can also regulate T2DM signaling through its interaction with TNF and PPARG (Figure 4C).

Several cytokines or peptides are Adipose tissue-derived adipokines (Figure 5D), HSC-derived stellakines (Figure 5E), muscle-derived myokines (Figure 5F), and hepatocyte-derived hepatokines participate in response to certain nutrition and/or physical activity conditions. Contracting skeletal muscle generates and releases a variety of cytokines and other peptides, which are collectively termed “myokines”. Myokine mediates muscle myogenesis and regeneration, and communicates with liver, adipose tissue, and pancreas (Table 3). Physical inactivity can change the production profile of myokines and their responses since the production of most myokines is affected by muscle contraction. The action of myokines for exercise-induced adaptation in skeletal muscle is responsible for oxidation and lipolysis of fatty acid and disposal of glucose (118).”

The authors apologize for this error and state that this does not change the scientific conclusions of the article in any way. The original article has been updated.

